# Generative pretraining for drug molecule design with bidirectional structure-property optimization

**DOI:** 10.1038/s42004-026-02093-5

**Published:** 2026-06-08

**Authors:** Yingying Song, Song He, Xiaochen Bo, Zhongnan Zhang

**Affiliations:** 1 School of Informatics, Xiamen University, Xiamen, China; 2 Department of Advanced & Interdisciplinary Biotechnology, Academy of Military Medical Sciences, Beijing, China

**Keywords:** Drug discovery, Cheminformatics

## Abstract

Designing drug-like molecules that satisfy both properties requirements and structural constraints remains challenging. Current generative approaches typically reduce properties to numerical constraints and treat molecular structures as deterministic functions of properties, failing to capture complex, nonlinear structure-property relationships and limiting generative diversity and controllability. Therefore, we propose BiSP-GP, a bidirectional structure-property generative pretraining framework that unifies molecular generation and property prediction as a single sequence modeling task. BiSP-GP serializes continuous properties into semantic token sequences for joint modeling with molecular structures in a shared sequence space. Molecular generation and property prediction are cast as autoregressive sequence modeling tasks, with a cross-modal decoder supporting bidirectional mapping. The framework also incorporates scaffolds as conditional inputs to guide structure generation. Experiments demonstrate that BiSP-GP achieves strong performance gconditional molecular generation, property prediction, and downstream tasks. A case study on PAK1 further validates the model’s generative ability and shows improved binding capacity in molecular docking evaluation.

## Abstract (web summary)

Designing drug-like molecules that satisfy both property requirements and structural constraints is a persistent challenge. Here, the authors introduce BiSP-GP, a bidirectional structure-property generative pretraining framework that unifies molecular generation and property prediction through shared autoregressive sequence modeling, enabling joint learning and bidirectional optimization of structure-property relationships, thereby enhancing controllable molecular generation while supporting molecular property prediction and candidate drug optimization tasks.

## Introduction

In the field of drug discovery, efficiently identifying high-quality candidate molecules that meet specific property requirements remains a core research objective^1–3^. Although traditional virtual screening techniques are mature and reliable, they are inherently constrained by the chemical diversity of existing molecular databases, limiting exploration to known chemical space and hindering the discovery of novel structural candidates with superior properties^4^. Recent breakthroughs in artificial intelligence offer innovative solutions to this challenge. Deep generative models leverage deep learning to extract latent structural distributions from large-scale molecular datasets and generate molecules through intelligent sampling in high-dimensional latent spaces, establishing a framework for de novo molecular design^5,6^. This data-driven approach not only overcomes the chemical-space limitations of traditional virtual screening but also enables target-directed molecular design based on specified property requirements, effecting a paradigm shift from “screening the known” to “creating the unknown”^7,8^.

For instance, models based on recurrent neural networks (RNNs)^9–11^, variational autoencoders (VAEs)^12,13^, generative adversarial networks (GANs)^14–16^, and graph neural networks (GNNs)^17,18^ have demonstrated strong performance in terms of molecular validity, diversity, and novelty. To enhance the controllability of molecular generation, researchers have developed various optimization strategies. On the one hand, techniques, such as reinforcement learning^6,19,20^ and Bayesian optimization^21,22^, enable targeted structural optimization for desired properties. On the other hand, conditional molecular generation models provide solutions for specific requirements. For example, conditional RNN-based models^11,23^, conditional VAE-based models^24–26^, and conditional graph-based generative models^27,28^ incorporate attribute labels and structural information as conditional constraints, thereby achieving directed generation of molecules with specified properties or structural features. The Transformer model, leveraging its powerful self-attention mechanism, has achieved groundbreaking success in natural language processing (NLP). This capability to effectively capture long-range dependencies has also driven significant innovations in molecular generation. Studies by Bagal et al.^29^ and Yang et al.^30^ applied the Transformer model to SMILES (simplified molecular input line entry system)^31^, feeding molecular properties or scaffold structures as conditional inputs to the decoder, thereby achieving preliminary control over target characteristics. These works demonstrated that Transformer-based models can guide multiple properties through conditional training, highlighting their potential for controllable molecular generation.

However, although enabling conditional control, these methods treat property information as superficial tokens without establishing deep semantic links between structures and properties. Consequently, such approaches lack semantic awareness of properties and fail to capture the true chemical significance of property values and their correspondence with molecular structures. To overcome this limitation, recent studies^32,33^ have explored joint modeling paradigms for molecular generation and property prediction, aiming to enhance the model’s understanding of target properties through multitask learning. This modeling paradigm enables the model to simultaneously capture the relationships between molecular structures and properties, thereby demonstrating strong potential across a range of practically relevant tasks, including molecular property prediction^34,35^ (e.g., toxicity prediction^36–39^, side-effect prediction^40^ and bioactivity prediction^41^), and property-constrained molecular design. However, although joint modeling approaches improve the utilization of property information to some extent, existing methods typically formulate molecular generation as an explicit deterministic function of property variables when handling continuous properties. Such an assumption is overly restrictive, as it introduces additional inductive bias and limits the model’s ability to capture complex, nonlinear structure-property relationships.

To address these challenges, we propose BiSP-GP, a bidirectional structure-property generative pretraining framework that unifies molecular design and property modeling within a single sequence modeling paradigm. Unlike conventional approaches that treat molecular properties as fixed numerical conditions or regression targets, BiSP-GP introduces a fine-grained property serialization strategy that decomposes continuous physicochemical attributes (e.g., QED, LogP and SAS) into structured semantic token sequences comprising symbols, digits, and positional encodings. This digit-level representation preserves numerical precision while transforming molecular properties into serializable linguistic expressions, allows them to be modeled alongside molecular structures within a unified generative language space. This design circumvents the inductive bias inherent in explicitly modeling structures as deterministic functions of properties.

Building on this representation, BiSP-GP establishes a unified sequence generation framework in which both molecule generation and property prediction are formulated as autoregressive sequence modeling tasks. Within this framework, molecular structures and property descriptions are treated as complementary modalities, and their bidirectional relationships are learned through a shared encoder and cross-modal interactions. This obviates the need for explicit regression heads and facilitates flexible modeling of complex nonlinear structure-property relationships.

Furthermore, by embedding both structure and property information into the same token space, we introduce a stochastic masking strategy during pretraining, where subsets of condition tokens are randomly masked. This design exposes the model to diverse combinations of incomplete and mixed conditions, thereby enhancing its robustness and generalization across varying constraint scenarios. Consequently, at inference time, BiSP-GP supports both controllable molecular generation and accurate prediction of molecular properties (QED, LogP, and SAS) within a unified framework. Different generation modes can be flexibly specified by adjusting input token prefixes, without requiring architectural modifications or task-specific fine-tuning.

Experimental results demonstrate outstanding performance across multiple dimensions. In conditional molecular generation, BiSP-GP consistently surpasses baseline models in both molecular quality and property-control accuracy across various settings. For property prediction, the model achieves notably high R² scores for key properties. Moreover, the learned molecular representations exhibit strong transferability in downstream tasks, achieving statistically supported improvements on multiple molecular benchmarks while maintaining robust overall performance. In a case study on PAK1 inhibitor optimization, BiSP-GP demonstrates its practical utility through scaffold-preserving analog generation, high-fidelity structure-property reconstruction, and property-guided lead optimization. These capabilities jointly enhance physicochemical properties and predicted binding affinity, thereby highlighting the model’s potential for real-world drug discovery.

## Results

### Model overview

**Fig. 1:**
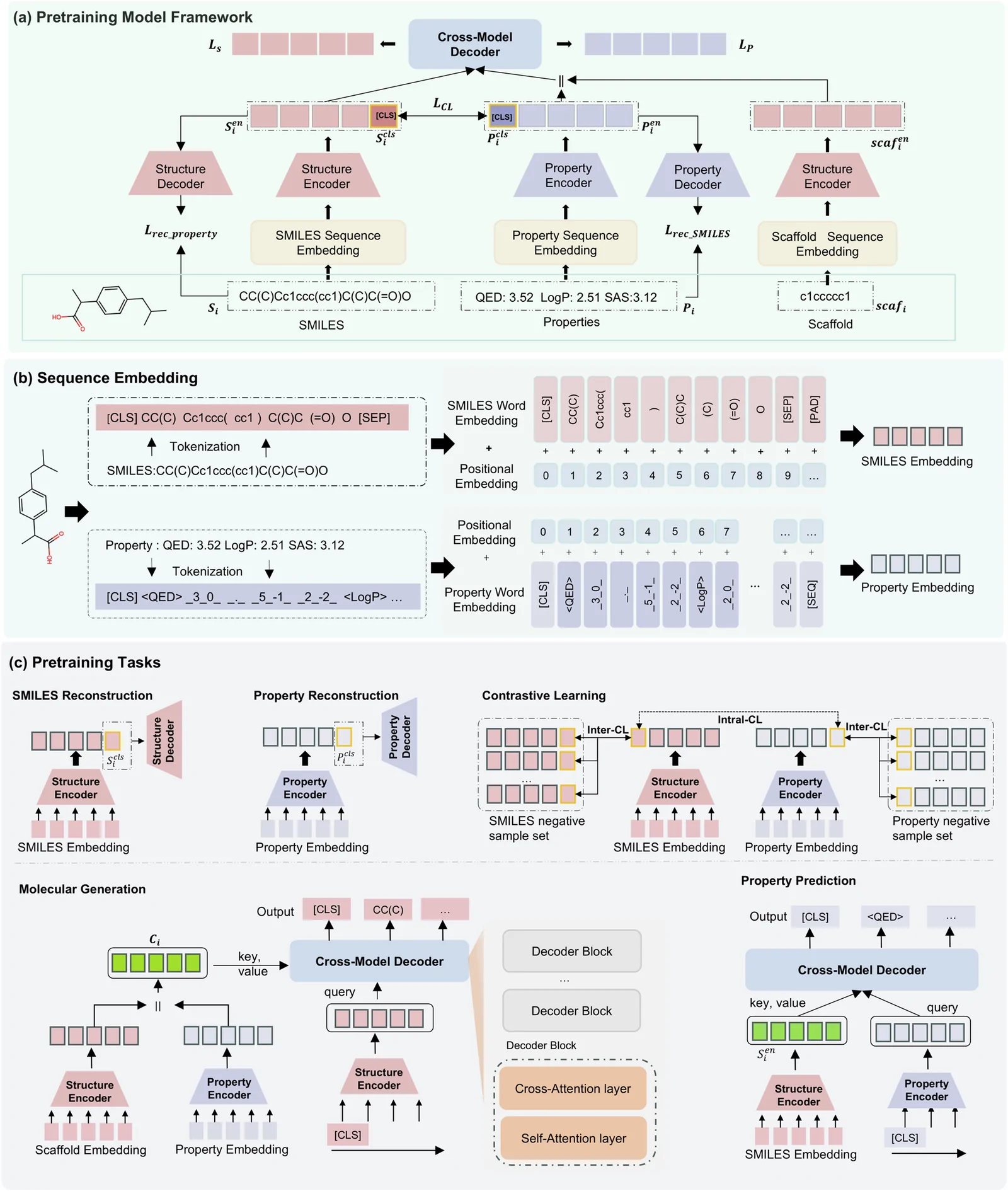
Overview of the BiSP-GP model framework, sequence embedding, and pretraining tasks. **a** Model framework: The framework includes dual autoencoders for processing SMILES sequences and property vectors respectively, along with a cross-modal decoder for fusing molecular SMILES with attribute information and enabling bidirectional modeling. **b** Feature embedding workflow: Molecular SMILES strings and continuous property values are serialized into discrete tokens, then transformed into structure and property embeddings through word embedding and positional encoding. **c** Five pretraining tasks: SMILES sequence reconstruction, property sequence reconstruction, contrastive learning for aligning molecular structure and property embedding spaces, multimodal condition-based molecule generation, and structure-based property prediction.

Figure 1a illustrates the overall framework of BiSP-GP, which is centered on dual autoencoders and a cross-modal decoder. The structure encoder processes SMILES or scaffold sequences, whereas the property encoder handles property sequences; both encoders are based on the Transformer architecture to capture long-range dependencies and contextual information within the sequence. The cross-modal decoder integrates latent features from both encoders and enables efficient inter-modal interaction and information transfer via a cross-attention mechanism.

During input processing (Fig. 1b), molecular SMILES and their associated continuous property values are first serialized into discrete symbol sequences. These sequences are subsequently processed through word embedding and positional encoding layers, respectively, ultimately producing context-aware molecular structure and property embeddings.

During pretraining (Fig. 1c), BiSP-GP employs the following strategies to enhance the modeling of molecular structures and properties. First, sequence embeddings of both modalities are passed into separate autoencoders for deep feature extraction. Second, intra-modal and inter-modal contrastive learning tasks are introduced to improve representation quality and cross-modal alignment. Finally, aligned multimodal features are processed by a unified cross-modal decoder via cross-attention for deep interaction, simultaneously achieving two tasks: (i) molecular structure modeling, which captures the underlying distribution of chemical space to support both unconditional generation and conditional generation based on property (and/or scaffolds), and (ii) property sequence modeling conditioned on molecular structures.

### Multi-scenario molecular generation with BiSP-GP

To comprehensively validate the molecular generation performance advantages of BiSP-GP, we designed a systematic evaluation framework spanning unconditional, single/multi-property conditional, scaffold-conditional, and property–scaffold joint generation tasks. The pretrained BiSP-GP model was used to autoregressively generate SMILES sequences according to given input conditions. The quality of all generated molecules was quantitatively assessed using validity (Val), uniqueness (Uni), novelty (Nov), and internal diversity (IntD) metrics. Additionally, mean absolute deviation (MAD) was employed to measure property control errors, while the scaffold similarity ratio (Sim_ratio) metric was used to quantify the retention accuracy of input scaffolds.

#### Evaluation of unconditional molecular generation

The vast chemical space cannot be exhaustively explored, highlighting the need for generative models capable of producing novel, valid, and diverse molecules. As an initial assessment, we evaluated BiSP-GP’s basic generation capability by generating 1000 molecules through an unconditional autoregressive task, comparing it against five representative baseline methods: RNN-based CharRNN^9^, GAN-based LatentGAN^42^, Transformer-based MolGPT^29^, SPMM^33^ and GP-MoLFormer^43^. BiSP-GP achieved the highest composite score (V*U*N*I) of 0.804, outperforming the other baseline methods (Table 1). In terms of validity, BiSP-GP closely matched the top-performing GP-MoLFormer, demonstrating robust modeling of molecular syntax. For uniqueness, BiSP-GP achieved 0.999, which is comparable to CharRNN and close to the perfect score of MolGPT, reflecting low duplication rates and broad structural coverage. Although LatentGAN and SPMM achieved 2.3% and 2.4% higher novelty respectively, both exhibited substantially lower diversity scores by 2.5% and 3.6%, revealing a high-novelty/low-diversity trade-off. In contrast, BiSP-GP simultaneously achieved high novelty and superior diversity, enabling balanced exploration of chemical space and generating a richer repertoire of candidate structures. As shown in Supplementary Fig. S1a–d, the generated molecules exhibit reasonable drug-like property distributions. Moreover, the mean nearest-neighbor Tanimoto similarity (Supplementary Fig. S1e) is 0.743, indicating that the generated molecules are structurally related to, yet meaningfully distinct from, the pre-training data within the learned chemical space.

**Table 1:** Comparison of the molecular generation quality between BiSP-GP and five baseline methods under unconditional constraints

Model	Val ↑	Uni ↑	Nov ↑	IntD ↑	V*U*N*I ↑
CharRNN*	0.975	0.999	0.842	0.856	0.702
LatentGAN*	0.897	0.997	0.949	0.857	0.727
MolGPT*	0.994	1.0	0.797	0.857	0.679
SPMM*	0.971	0.991	0.950	0.846^a^	0.773
GP-MoLFormer*	1.0	0.997	0.390	0.866	0.337
BiSP-GP (Ours)	0.986	0.999	0.926	0.882	0.804

"V*U*N*I" denotes a composite score computed as the product of validity (V), uniqueness (U), novelty (N), and internal diversity (I). Asterisk (*) indicates that the baseline results were taken from the original publications, where the results of CharRNN, LatentGAN, and MolGPT were reported in the MolGPT paper, and those of SPMM and GP-MoLFormer were reported in their respective papers. Results marked with "a" are unofficial and were reproduced by using the official model checkpoints.

#### Evaluation of property-conditioned molecular generation

**Fig. 2:**
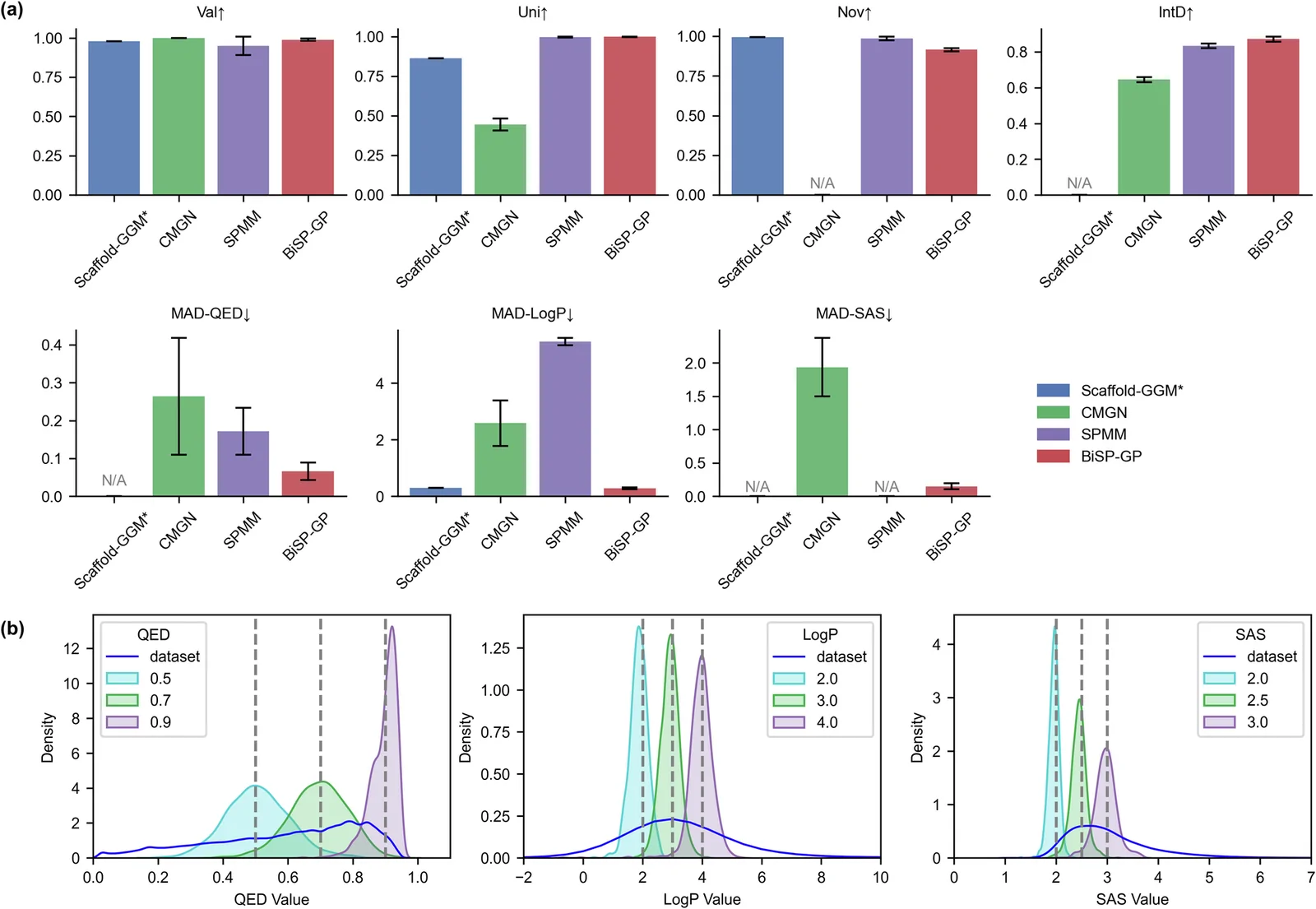
Performance evaluation of BiSP-GP for molecule generation under single-property constraints. **a** Comparison results of BiSP-GP with three baseline models in single-property constrained generation. Results marked with an asterisk (*) are directly taken from the original publications, while the others are reproduced using the released checkpoints of the baseline models. All baseline models had been trained with the target property to ensure fair comparison in single-property constrained generation. **b** Distributions of QED, LogP, and SAS for molecules generated by BiSP-GP under single-property constraints. The solid blue line represents the distribution of each property in the pretraining dataset, while the gray vertical dashed line indicates the target property value.

In pharmaceutical R&D, precise control of molecular properties is critical for designing high-quality candidate compounds. For example, in central nervous system drugs, LogP must remain within 1–3 to avoid nonspecific tissue distribution and metabolic instability^44^. To systematically evaluate the performance advantages of BiSP-GP in single-property control, we conducted property-constrained generation experiments targeting QED (set to 0.5, 0.7, 0.9), LogP (set to 2, 3, 4), and SAS (set to 2, 2.5, 3). For each target property value, BiSP-GP generated 1000 candidate molecules, and was compared against three representative baseline models: the classical conditional molecular generation method CMGN^45^, the scaffold-constrained generation model Scaffold-GGM^17^, and the multimodal generation framework SPMM^33^. Figure 2a shows that BiSP-GP achieved the lowest MAD values across all three properties, demonstrating excellent regulation accuracy (full comparison results are provided in Supplementary Table S1). Figure 2b further provides intuitive visualization results, with QED, LogP, and SAS distributions all centered on the target values and clearly diverging from the pretraining set distribution (blue line). BiSP-GP also outperformed all baselines in uniqueness, and IntD, demonstrating broad structural exploration under strict property constraints. Although its novelty was slightly lower than that of Scaffold-GGM and SPMM, BiSP-GP achieved a better overall balance among accuracy, diversity, and uniqueness.

**Fig. 3:**
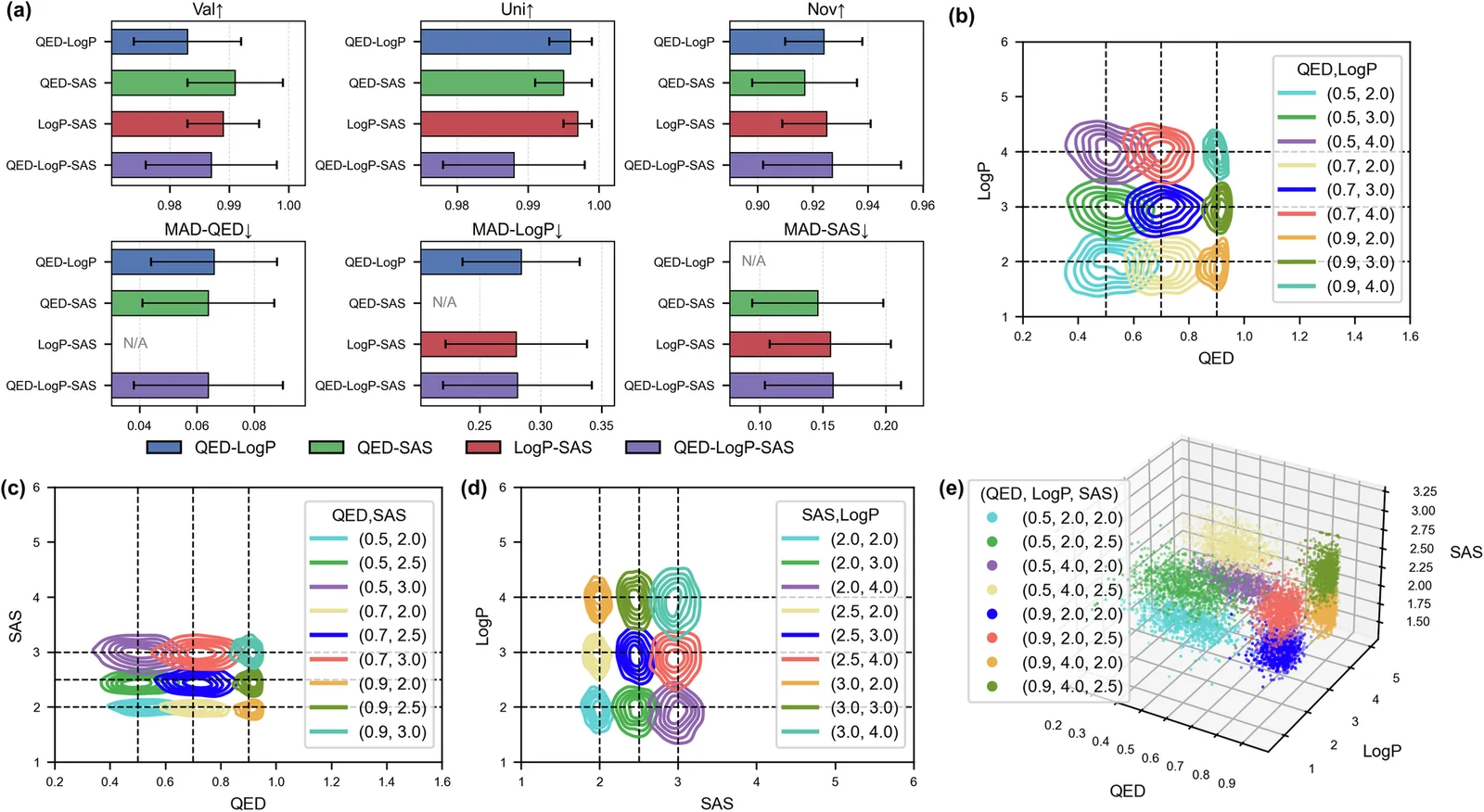
Performance evaluation of BiSP-GP for molecule generation under multi-property constraints. **a** BiSP-GP’s performance under four types of multi-property constraints, including QED-LogP, QED-SAS, LogP-SAS, and QED-LogP-SAS combinations. The results for each constraint type represent average performance under multiple sets of target attribute values. **b**–**e** Property distributions of molecules generated by BiSP-GP under four multi-property constraints. Dashed lines represent the set target property values, with horizontal, vertical, and depth axes corresponding to different properties.

**Fig. 4:**
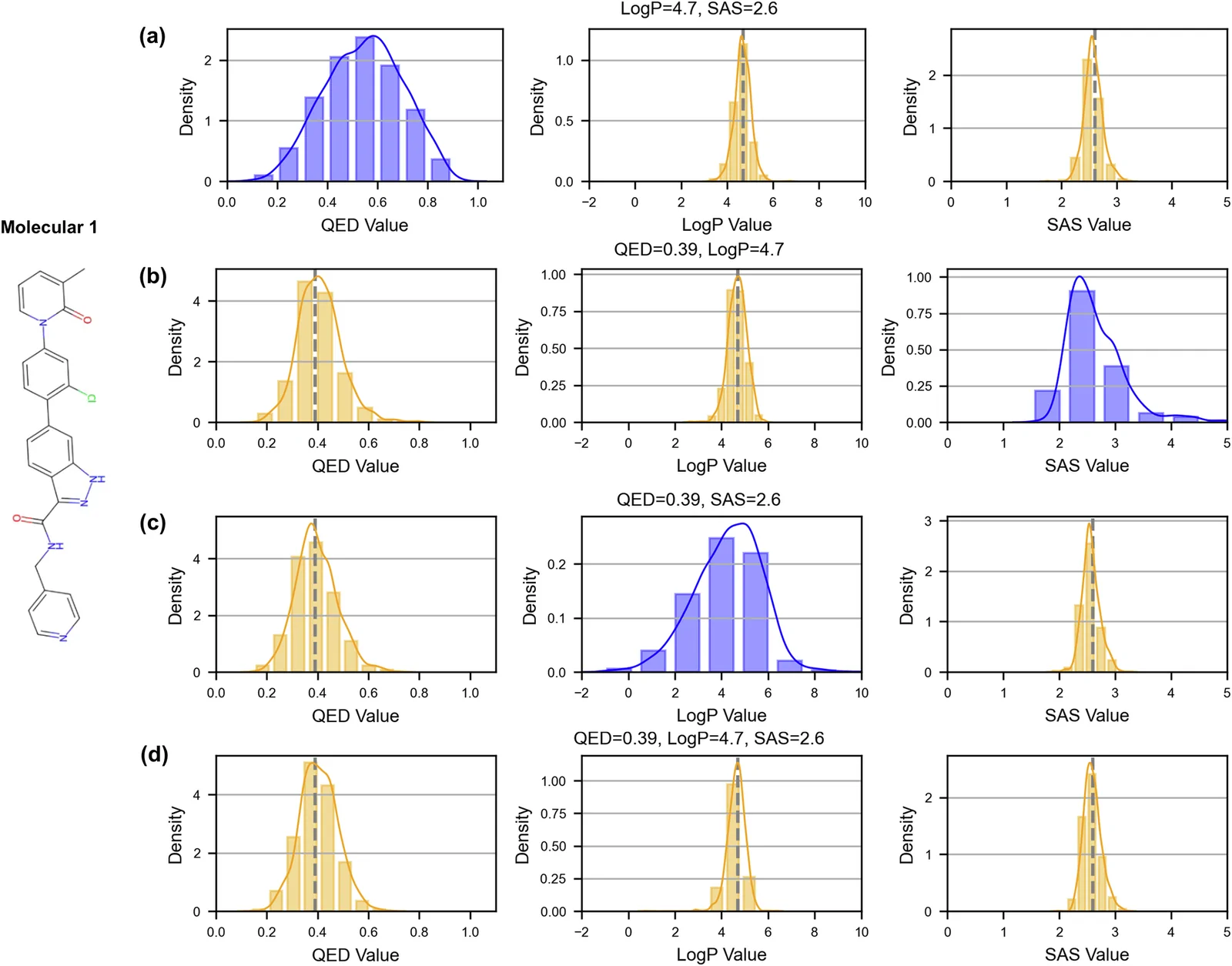
Property distributions of molecules generated by BiSP-GP under multiple property combinations derived from “Molecule 1”. **a** LogP = 4.7, SAS = 2.6; **b** QED = 0.39, LogP = 4.7; **c** QED = 0.39, SAS = 2.6; and **d** QED = 0.39, LogP = 4.7, SAS = 2.6. Vertical dashed lines mark the input target constraint values; yellow curves represent kernel density estimates (KDEs) of controlled properties of generated molecules, while blue curves show distributions of the uncontrolled properties.

Since real-world drug design requires simultaneous optimization of multiple properties^44,46^, we evaluated BiSP-GP in multi-property constrained generation. Figure 3a shows that the model achieved validity, uniqueness, and novelty scores above 0.9 in all test scenarios, confirming its stable structural generation capability under simultaneous constraints (detailed results are provided in Supplementary Table S2). The low MAD values, together with the distributions tightly clustered around target property values in Fig. 3b–e, jointly validate the model’s precise capability for coordinated multi-property control. To simulate a realistic drug design scenario, we used a real compound (“Molecule 1” in Fig. 4; QED = 0.39, LogP=4.7, SAS = 2.6) as input. Under this setting, we tested four property combinations, including three pairwise control settings and one triple-control setting, with 1,000 candidate molecules generated for each group. The results show (Fig. 4) that in all cases, the distribution patterns of each controlled property were determined by the specified input values, while uncontrolled properties retained their natural broad distributions. Notably, even under triple-property control, the model achieved high-precision matching of all target properties. These findings confirm the BiSP-GP’s ability to handle the complex interdependencies among multiple properties.

#### Evaluation of Scaffold-conditioned molecular generation

**Fig. 5:**
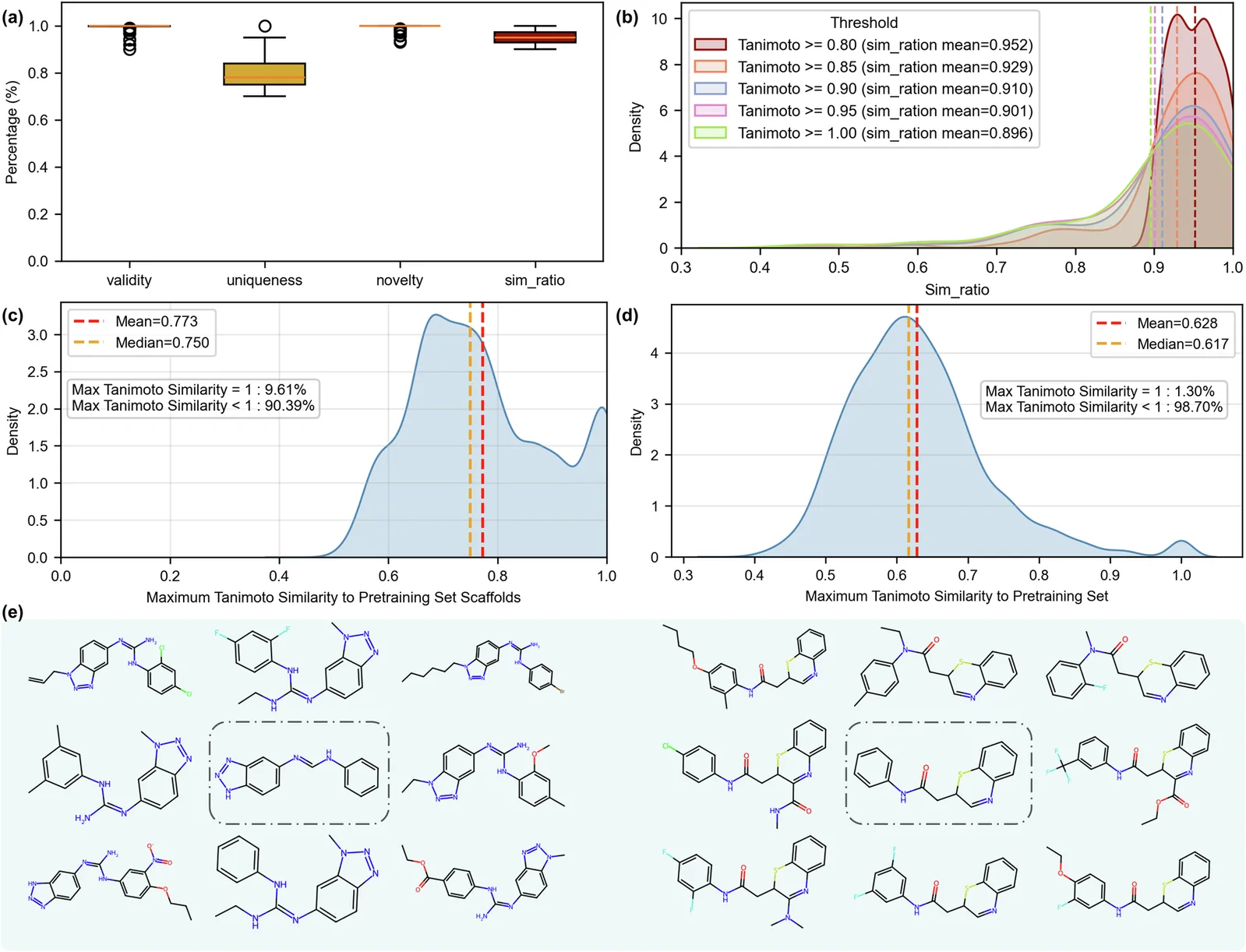
Performance evaluation, similarity analysis, and representative molecule generation of BiSP-GP under scaffold constraints. **a** Distribution of validity, uniqueness, novelty, and scaffold similarity ratio (sim_ratio) for BiSP-GP-generated molecules under constraints from 100 scaffolds absent from the pre-training dataset. **b** Distribution of scaffold similarity ratios under different Tanimoto similarity thresholds of 0.80, 0.85, 0.90, 0.95, and 1.00. The vertical dashed line indicates the mean sim_ratio. **c** Maximum scaffold similarity between generated molecules and training scaffolds after excluding exact duplicates. 9.61% of molecules have similarity = 1.0; 90.39% have similarity<1.0. **d** Maximum Tanimoto similarity between generated molecules and their nearest neighbors in the pretraining set. 1.30% of molecules have similarity=1.0; 98.70% have similarity<1.0. **e** Representative generated molecules using scaffolds from “Molecule 2” (left) and “Molecule 3” (right). Dashed boxes indicate the input scaffolds; remaining structures are the corresponding generated molecules.

Previous experiments validated BiSP-GP’s ability to generate molecules meeting target properties under property-guided conditions. In molecular optimization studies, researchers often aim to precisely tune molecular properties while preserving a specific pharmacophore scaffold^12^. To evaluate BiSP-GP’s scaffold-preserving generation capability, we tested 100 unseen scaffolds outside the pretraining set, generating 1000 SMILES per scaffold. As shown in Fig. 5a, BiSP-GP achieves strong generative performance across unseen scaffolds, with validity and novelty consistently above 0.8 and 75% of test cases achieving uniqueness scores greater than 0.75. The ${Sim\_ratio}$ of all generated molecules exceeds 0.8, indicating effective preservation of the input scaffold. Consistently, Fig. 5b shows that scaffold retention remains high across different Tanimoto thresholds (0.80-1.00), confirming the model’s ability to maintain scaffold structures during generation. To more intuitively illustrate BiSP-GP’s scaffold-guided generation capability, Fig. 5e presents generation examples for two specific scaffolds (“Molecule 2“and “Molecule 3”).

To further investigate the source of differences between generated molecules and the pretraining data, we examined the maximum Tanimoto similarity at two levels: scaffold and whole-molecule. At the scaffold level (Fig. 5c, the mean maximum similarity to the pretraining set is 0.773, and 90.39% of scaffolds are novel, indicating substantial divergence. At the whole-molecule level (Fig. 5d), the mean maximum similarity further decreases to 0.628. Since scaffold-level differences already exist, this additional reduction can be attributed to side-chain variations. Taken together, these results indicate that the differences originate from both the use of out-of-distribution scaffolds and the side-chain modifications built upon them.

#### Evaluation of Scaffold-property jointly conditioned molecular generation

**Fig. 6:**
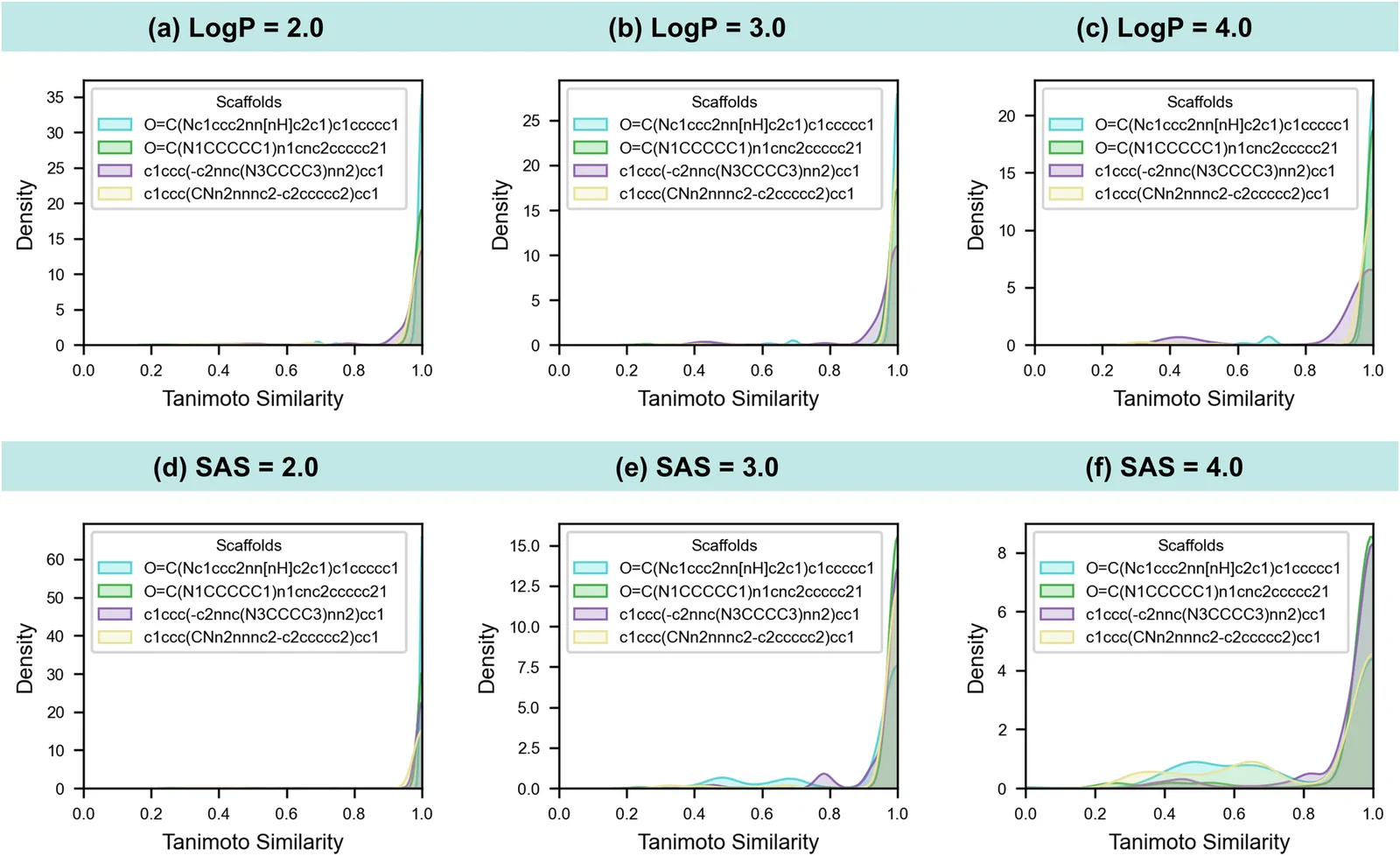
Tanimoto similarity distributions between the scaffolds of molecules generated by BiSP-GP and the corresponding input scaffolds under single-property and scaffold constraints. **a**–**c** Distributions of Tanimoto similarity between scaffolds of generated molecules and input scaffolds under different LogP values (2.0, 3.0, 4.0) with scaffold constraints. **d**–**f** Distributions of Tanimoto similarity between scaffolds of generated molecules and input scaffolds under different SAS values (2.0, 3.0, 4.0) with scaffold constraints. In each subplot, the x-axis represents Tanimoto similarity, and the y-axis represents density. Different colored density plots correspond to different input scaffolds, showing the kernel density distributions of similarity between generated molecules and their respective input scaffolds; higher peaks indicate greater density of generated molecules near that similarity value.

**Fig. 7:**
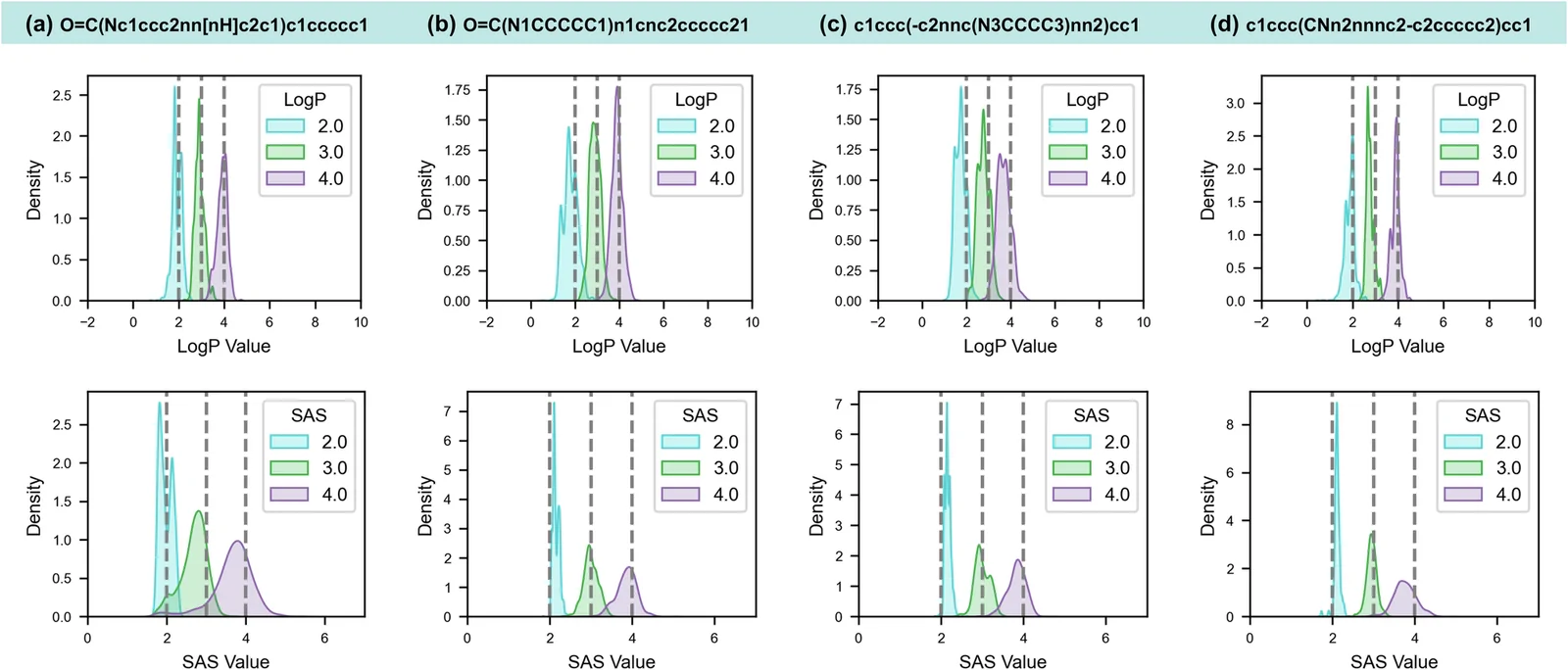
Property distributions of molecules generated by BiSP-GP under single-property and scaffold constraints. **a**–**d** Each column corresponds to one input scaffold, showing the property distributions of molecules generated under LogP and SAS targets for each scaffold. In each subplot, different colored kernel density plots represent the property distributions of molecules generated under the corresponding scaffold constraint and property target. Higher peaks indicate a larger number of molecules generated near that property value. The x-axis represents LogP or SAS values, the y-axis represents density, and vertical dashed lines indicate the target property values.

We further evaluated BiSP-GP’s ability to generate molecules satisfying multiple property constraints under fixed-scaffold conditions. This task simulates real-world drug optimization, where compounds are generated from a known fragment while meeting specified property ranges. Four representative test scaffolds with diverse aromatic and heterocyclic combinations were tested under constraints from single-property (LogP or SAS) to multi-property (LogP-SAS or QED-LogP-SAS). For each combination, multiple target values were set, with 1000 molecules generated per target. Figures 6 and 7 show two-dimensional evaluation of scaffold- and single-property-constrained generation. The Tanimoto similarity distribution between generated molecules and target scaffolds exhibits a pronounced peak near 1.0, indicating strongly preservation of the input scaffold structure (Fig. 6). Figure 7 further shows that the LogP and SAS values of the generated molecules are tightly clustered around the designated target values, confirming the model’s precise control over specific properties. Under scaffold- and multi-property conditions (Supplementary Figs. S2 and S3), most generated molecules still maintain high structural consistency with the input scaffolds. Although property distributions under multi-property constraints are more dispersed than those under single-property control, the generated molecules still form clear clusters within designated ranges and remain overall concentrated near the target values. Overall, BiSP-GP achieves multi-property co-optimization while preserving scaffold stability, highlighting its ability to balance structure preservation and multi-property optimization.

### Case study for Scaffold-preserving lead optimization of a PAK1 inhibitor

To evaluate the practical utility of BiSP-GP in realistic lead optimization scenarios, we selected a literature-reported PAK1 (p21-activated kinase 1) inhibitor (“Molecule 1”) as a case study^47^. PAK1, a serine/threonine kinase, is implicated in cancers and neurodegenerative diseases, making its inhibitors a key focus of drug research. We designed three generation settings to systematically assess the model’s behavior under progressively stricter conditions.

**Fig. 8:**
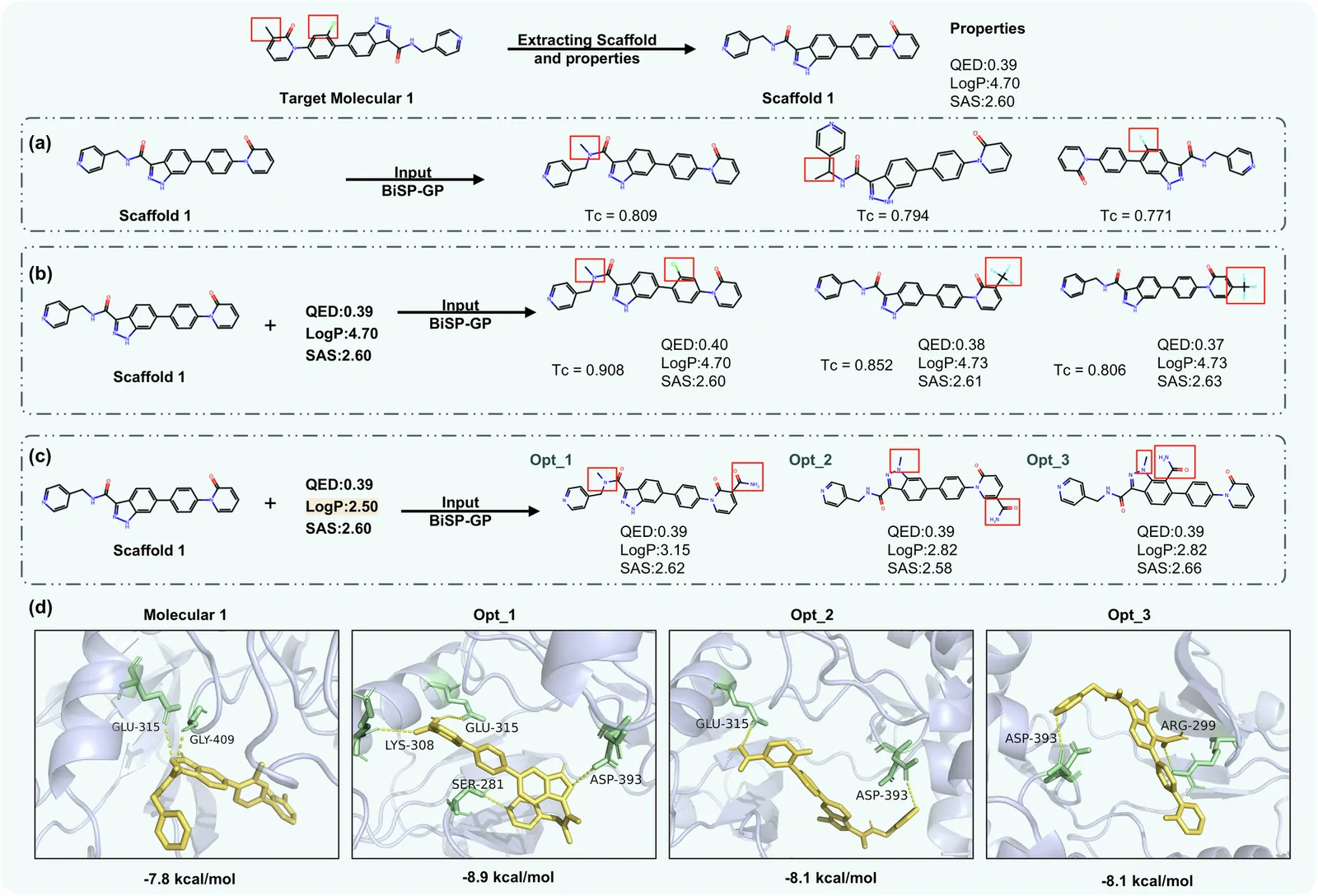
Scaffold-preserving lead optimization of a PAK1 inhibitor using BiSP-GP under scaffold and multi-property constraints with docking analysis. **a** Molecule generation under scaffold-only constraints. Tc denotes the Tanimoto similarity between generated molecules and the reference compound. **b** Molecule generation under joint scaffold and property constraints (QED = 0.39, LogP = 4.70, SAS = 2.60). **c** Molecule generation under scaffold and multi-property constraints with a reduced target LogP (2.50) while maintaining QED and SAS. Optimized molecules (Opt_1 - Opt_3) are displayed. **d** Molecular docking poses of “Molecule 1” and optimized molecules (Opt_1 - Opt_3) in the PAK1 binding site (PDB ID: 4EQC), along with their corresponding docking scores, with key interacting residues highlighted.

In the first setting, only the core scaffold of Molecule 1 was provided as a structural constraint. This experiment evaluates whether BiSP-GP can generate chemically valid analogs while preserving key pharmacophoric elements. Among the valid generated molecules, the three candidates with the highest Tanimoto similarity to Molecule 1 were selected as representative examples. As shown in Fig. 8a, the generated molecules retained the essential scaffold framework and exhibited diverse side-chain modifications, demonstrating reliable scaffold-conditioned generation capability.

In the second setting, the scaffold constraint was combined with the original physicochemical properties of Molecule 1 as multimodal conditions. This configuration aims to assess the model’s ability to perform high-fidelity analog reconstruction under joint structure-property constraints. The top generated candidate achieved a Tanimoto similarity of 0.908 relative to Molecule 1 (Fig. 8b). This high similarity indicates that BiSP-GP effectively performs local structural refinement and lead analog generation when guided by the reference property profile.

In the third setting, the scaffold was fixed while optimized physicochemical targets were specified. Given the lipophilicity of Molecule 1 (LogP = 4.70), which may compromise drug-likeness, the target LogP was reduced to 2.50 while QED and SAS values were held constant. Molecular docking of the PAK1 kinase domain (PDB ID: 4EQC) with candidate molecules that preserve the core scaffold yielded an average docking score of −8.15 kcal/mol, which is approximately 0.35 kcal/mol lower than that of the reference molecule (−7.8 kcal/mol). The three molecules exhibiting the most favorable docking scores were selected as optimized representatives (Opt_1 - Opt_3). As shown in Fig. 8c, these candidates preserve the core scaffold while incorporating more polar side chains, resulting in reduced lipophilicity (LogP = 3.15, 2.82, and 2.82), while maintaining favorable QED and SAS profiles. Figure 8d provides a visualization of their binding modes, revealing that these candidates not only retain the critical hydrogen-bond interaction with GLU-315 but also form additional polar interactions with residues including ASP-393, SER-281, and LYS-308. Notably, ASP-393 has been reported to play a key role in PAK1 catalytic activity and to participate in ligand interactions within the binding pocket^48^, supporting the biological plausibility of the observed interactions.

Collectively, these results demonstrate that BiSP-GP is capable of scaffold-preserving lead generation under diverse constraint settings, enabling controlled optimization of physicochemical properties while maintaining docking performance comparable to that of the reference molecule.

### Evaluation of SMILES-to-property modeling and transferability of BiSP-GP

#### Evaluation of SMILES-to-property sequence modeling

Similar to SMILES generation, BiSP-GP can generate property sequences from SMILES. Although these properties can be directly computed with RDKit, the key objective is to assess whether the model can independently learn the structure-property relationships and generate syntactically valid and numerically accurate property descriptions. We randomly selected 1000 unseen molecules, used only their SMILES strings as input, and generated property sequences through autoregression. All output property sequences maintain strict grammatical legality and can be accurately converted into numerical vectors, verifying the reliability and parsability of the string-based representation. The predicted values for LogP, QED, and SAS achieved R² scores of 0.999, 0.997, and 0.987, respectively, showing excellent agreement with ground-truth values, with data points densely distributed along the y=x diagonal (Supplementary Fig. S4a–c). To further evaluate the model’s robustness against syntactic variation, we conducted robustness testing using 1000 randomized SMILES (Supplementary Fig. S4d–f)). The results indicate that BiSP-GP remains reliable under non-standard inputs: the LogP prediction error (RMSE = 0.1382) resides within the experimental uncertainty range^49^, while the QED and SAS errors constitute only about 5% of their respective value range. These results highlight BiSP-GP’s accuracy in modeling structure-property relationships and the effectiveness of string-based representations, providing a strong foundation for unified frameworks in molecular design and property prediction.

#### Downstream task transfer evaluation

**Fig. 9:**
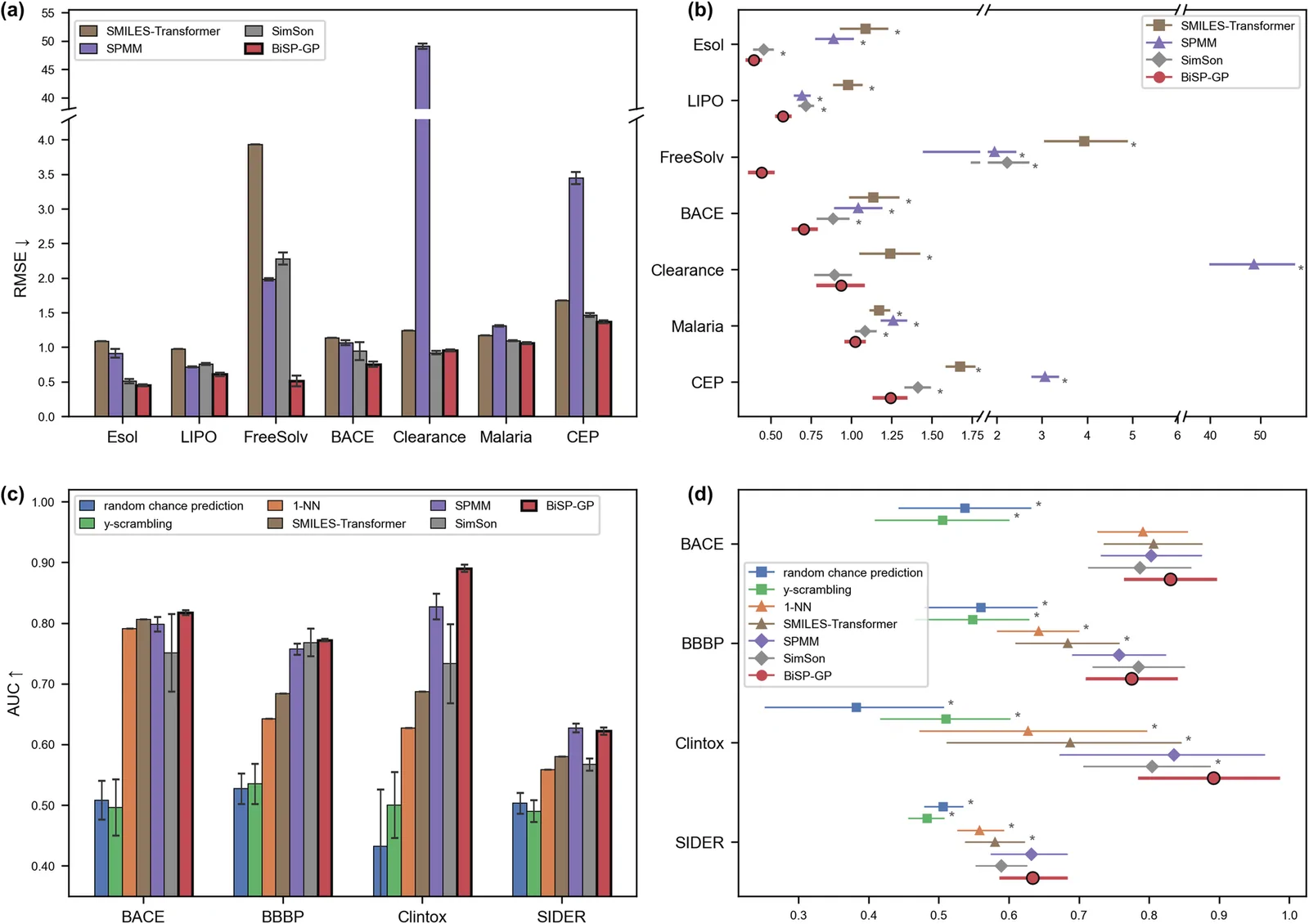
Performance comparison between BiSP-GP and baseline models on classification and regression downstream tasks. **a**, **c** Regression and classification performance, measured by RMSE and AUC, respectively. For each task, all models were trained using four random seeds, and results are reported as mean ± standard deviation. **b**, **d** Regression and classification performance based on averaged predictions across random seeds, with 95% confidence intervals. Predictions (or predicted probabilities for classification) from different random seeds are averaged at the sample level. For regression tasks, bootstrap resampling was used to estimate confidence intervals and assess statistical significance; for classification tasks, DeLong’s method was applied. Points represent RMSE (**b**) or AUC (**d**), horizontal lines indicate 95% confidence intervals, and asterisks (*) denote statistically significant differences (*p* < 0.05).

To further evaluate the representation learning capability and cross-task transferability of BiSP-GP, the pretrained structure encoder was used as a fixed feature extractor and combined with a lightweight prediction head. The model was evaluated on nine MoleculeNet^50^ datasets, as well as two additional datasets, Malaria^51^ and CEP^52^ (Fig. 9, and Supplementary Tabless S3–S6).

For regression tasks (Fig. 9a, b), BiSP-GP achieves the lowest mean RMSE on six datasets, ESOL, LIPO, FreeSolv, BACE, Malaria, and CEP, relative to SMILES-Transformer^53^, PretrainGNN^54^, SPMM^33^ and SimSon^55^. Statistical analysis indicates that the improvements over SMILES-Transformer and SPMM are significant across all tasks. Although SimSon attains a marginally lower RMSE on the Clearance dataset, the difference is not statistically significant (*p* = 0.20). Moreover, BiSP-GP exhibits generally narrower 95% confidence intervals, suggesting improved prediction stability.

For the classification task, in addition to the four main models, we incorporated supplementary baselines, including random chance prediction and 1-nearest neighbor (1-NN) models, to facilitate a more comprehensive evaluation. Furthermore, to assess the reliability of BiSP-GP, we conducted y-scrambling, wherein the model was fine-tuned using randomly permuted labels. As illustrated in Fig. 9c, d, BiSP-GP achieves the highest mean AUC on the BACE, BBBP, and Clintox datasets. Statistical analysis based on DeLong’s test^56^ further indicates that BiSP-GP significantly outperforms SMILES-Transformer and the supplementary baselines on the BBBP, Clintox, and SIDER datasets. On the BBBP dataset, BiSP-GP attains an AUC of 0.775, which is comparable to that of SimSon (0.785), with no statistically significant difference. Moreover, the y-scrambling results yield AUC values approximating random performance across all datasets. This observation confirms that the predictive performance of BiSP-GP arises from genuine structure-property relationships, rather than from spurious correlations or label artefacts.

To further investigate the influence of dataset overlap, we quantified the molecular overlap between the pretraining dataset and downstream splits (Supplementary Fig. S5). The extent of overlap varies considerably across datasets, ranging from negligible in CEP (0.033%) to substantially higher in ESOL and FreeSolv. Despite this variation, BiSP-GP achieves consistent performance across datasets, suggesting that the observed improvements are unlikely to be primarily attributable to dataset overlap. Rather, they reflect the model’s ability to acquire generalizable molecular representations during pretraining.

## Discussion

The proposed BiSP-GP model achieves bidirectional molecular structure-attribute modeling through attribute linguistic modeling and cross-modal contrastive learning. To assess the contribution of these key components, we designed two ablated variants, ${{\mbox{BiSP}}-{\mbox{GP}}}_{{\mbox{value emb}}}$, which replaces property serialization with numerical vector embeddings, and BiSP-GP_w/oCL_, which removes the contrastive learning module. We systematically evaluated these variants across three tasks. The first task was conditional molecular generation, including unconstrained generation, nine single-property conditions (QED = 0.5/0.7/0.9, LogP = 2/3/4, SAS = 2/2.5/3), and 54 multi-property combinations derived from them. Each variant generated 1000 candidate molecules per condition, with final performance calculated as the average across all conditions. The second task involved predicting QED, LogP, and SAS properties for 1000 unseen SMILES molecules. The third task comprised downstream property prediction, including nine MoleculeNet subtasks and two external datasets (Malaria and CEP).

Experimental results demonstrate that BiSP-GP outperforms both variants on nearly all metrics (Supplementary Tables S7 and S8). Compared to the full model, the ${{\mbox{BiSP}}-{\mbox{GP}}}_{{\mbox{value emb}}}$ variant shows increased MAD for LogP by 0.553 and 0.506 under single-property and multi-property conditions, respectively. Similarly, significant differences are observed in errors for QED and SAS. In property prediction task, BiSP-GP achieves substantially higher ${R}^{2}$ values than ${{\mbox{BiSP}}-{\mbox{GP}}}_{{\mbox{value emb}}}$, confirming that property serialization is crucial not only for conditional generation but also for enhancing structure-to-property prediction performance. Likewise, BiSP-GP_w/oCL_ leads to comprehensive degradation in generation quality and property controllability. Taking QED as an example, BiSP-GP_w/oCL_ achieves MAD values of 0.086 and 0.085 for single-property and multi-property predictions respectively, both higher than the full model by about 0.020. For LogP and SAS, errors increase by more than 0.04, while ${R}^{2}$ scores in property prediction decrease significantly. These results highlight the critical role of contrastive learning in enhancing cross-modal consistency and predictive accuracy. For downstream tasks (Supplementary Table S9), BiSP-GP achieved the best average performance among the compared methods in 8 out of 11 tasks (including four classification and seven regression tasks), indicating its effectiveness in supporting generation quality, property controllability, and multi-task generalization.

BiSP-GP demonstrates strong performance in conditional molecular generation, property prediction, and scaffold-preserving lead optimization. In this study, molecular docking was employed as an initial structure-based evaluation tool to assess the plausibility of ligand-target interactions. However, docking alone is insufficient to fully characterize pharmacological efficacy, selectivity, metabolic stability, or safety profiles. In addition, although SAS was included as a controllable physicochemical property to demonstrate multi-property controllability, it remains a computational descriptor derived from rule-based and statistical information, and therefore cannot fully capture real synthetic feasibility. Therefore, future work incorporating more advanced synthesis feasibility assessment strategies, together with experimental validation, and more comprehensive pharmacological evaluation, will be important for supporting the practical applicability of the generated molecules in real-world drug discovery scenarios.

## Methods

### Embedding of molecular SMILES and properties sequences

BiSP-GP employs a dual-modal sequence embedding strategy, representing molecular information as SMILES and property sequences (Fig. 1b). The model first tokenizes both types of inputs. For SMILES, it adopts a byte pair encoding (BPE)-based^57^ subword tokenization to decompose SMILES into chemically meaningful substructure. This reduces sequence length and improves capture of local semantics. For continuous properties, inspired by the numerical encoding strategy^58,59^, a structured discretization method is applied to decompose each property value into semantic units, including property identifiers, sign, integer parts, decimal point, and fractional parts. Each digit is combined with its numerical place value to form discrete tokens. For example, “QED = 0.23” is converted into the sequence “<QED> _-_ _0_0_ _._ _2_-1_ _3_-2_“ (Implementation details can be found in the Supplementary Note 1). All sequences are further augmented with [CLS] and [SEP] tokens at the beginning and end, respectively, to indicate sequence boundaries.

Subsequently, the model learns embedding representations from the tokenized sequences. Taking SMILES as an example, the tokenized sequence is denoted as ${S}_{i}=\{{s}_{0},\,{s}_{1},\ldots {s}_{N}\}$, with the corresponding positional indices defined as ${S}_{i}^{{\mathrm{pos}}}=\left\{{\mathrm{0,1}},\ldots ,N\right\}$, where N denote the length of the SMILES sequenc99es. A learnable word embedding layer ${E}_{{\mathrm{word}}}^{s}$ and a positional embedding layer ${E}_{{\mathrm{pos}}}^{s}$ are employed to map discrete tokens and positional information into continuous vectors, which are then combined to form the SMILES sequence embedding:

1 $${E}_{{\mathrm{seq}}}^{s}\left({S}_{i}\right)={E}_{{\mathrm{word}}}^{s}\left({S}_{i}\right)+{E}_{{\mathrm{pos}}}^{s}\left({S}_{i}^{{\mathrm{pos}}}\right)$$

This joint embedding enables the model to capture both local chemical semantics and global sequential dependencies. The property sequence ${P}_{i}=\{{p}_{0},\,{p}_{1},\ldots {p}_{M}\}$ which denotes the discretized property tokens, undergoes the same embedding process as SMILES, resulting in the embedding representation ${E}_{{\mathrm{seq}}}^{P}\left({P}_{i}\right)$, where M denote the lengths of the property sequences.

To enhance the robustness and generalization of BiSP-GP, we introduce a stochastic masking mechanism on the property sequences. With a probability of 50%, all tokens of an entire property are replaced with the special symbol [UNK], thereby simulating scenarios with missing or incomplete property information. This strategy not only reflects real-world scenarios where certain properties are unavailable, but also serves as a data augmentation technique, effectively mitigating overfitting to specific properties and improving prediction performance for unseen molecular combinations.

### Pretraining tasks

During the model pretraining phase, we designed five self-supervised pretraining tasks (Fig. 1c) to deeply explore the intrinsic relationship between SMILES sequences and molecular properties, while improving the model’s generalization capability in molecular representation learning. These tasks include, (1-2) SMILES and property reconstruction tasks; (3) contrastive learning task; (4) molecule generation task and (5) property prediction task.

*The SMILES and property reconstruction tasks* are designed to enhance the model’s understanding of molecular structures and properties. For a training molecule i, the SMILES embedding representation ${E}_{{\mathrm{seq}}}^{s}\left({S}_{i}\right)$ and the property embedding representation ${E}_{{\mathrm{seq}}}^{P}\left({P}_{i}\right)$ are processed by separate Transformer encoders. Deep features are extracted via stacked multi-head self-attention layers, yielding the encoded representations ${S}_{i}^{{en}}$ and ${P}_{i}^{{en}}$, respectively. Since the [CLS] token output from each encoder aggregates global information of its corresponding modality through the self-attention mechanism, we use its encoded feature as the global semantic vector of that modality, where ${S}_{i}^{{cls}}$ represents the molecular structural features and ${P}_{i}^{{cls}}$ represents the molecular property features. During the SMILE reconstruction task, an autoregressive generation strategy is employed, using the global semantic vector ${S}_{i}^{{cls}}$ as conditional guidance to sequentially predict each token in the original sequence, the loss function is defined as:

2 $${L}_{{rec\_smiles}}={\sum }_{i=1}^{B}{\sum }_{n=0}^{N}\,H\left({y}_{n}^{s},{P}^{s2s}\left({s}_{n}|{s}_{0:n-1},{S}_{i}^{{cls}}\right)\right)$$

Here, $H\left(\cdot ,\cdot \right)$ denotes the cross-entropy function. ${P}^{s2s}\left({s}_{n}|{s}_{0:n-1},{S}_{i}^{{cls}}\right)$ represents the predicted probability distribution of the n-th token given the preceding tokens 0~(n−1) of ${S}_{i}$ and ${S}_{i}^{{cls}}$. ${y}_{n}^{s}$ denotes the one-hot encoding of the n-th ground-truth token of the SMILES sequence, and B is the batch size.

Similarly, the property reconstruction task employs ${P}_{i}^{{cls}}$ to guide the stepwise recovery of property tokens, with the corresponding reconstruction loss defined as:

3 $${L}_{{rec\_property}}={\sum }_{i=1}^{B}{\sum }_{m=0}^{M}\,H\left({y}_{m}^{p},{P}^{p2p}\left({p}_{m}|{p}_{0:m-1},{P}_{i}^{{cls}}\right)\right)$$

Here, ${P}^{p2p}\left({p}_{m}|{p}_{0:m-1},{P}_{i}^{{cls}}\right)$ denotes the probability distribution of predicting the next property token given ${p}_{0:m-1}$ and ${P}_{i}^{{cls}}$. ${y}_{m}^{p}$ denotes the one-hot encoding of the *m*-th ground-truth target token of the property sequence.

*We introduce a SMILES-property contrastive learning task* to enhance the model’s ability to capture cross-modal associations between molecular structures and properties. The task integrates both inter-modal contrastive learning (SMILES→Property, s2p; Property→SMILES, p2s) and intra-modal contrastive learning (SMILES ↔ SMILES, s2s; Property↔Property, p2p). Specifically, inter -modal contrastive learning aims to align molecular SMILES representations with their corresponding property embeddings in a unified semantic space, thereby promoting cross-modal semantic consistency. In contrast, intra-modal contrastive learning enhances discriminability within individual modality by encouraging structurally or property-similar samples to be positioned closer in the embedding space. For implementation, we design two independent linear projection heads, ${h}_{S}(\cdot )$ for SMILES and ${h}_{P}(\cdot )$ for properties, to map the global representations ${S}_{i}^{{cls}}$ and ${P}_{j}^{{cls}}$ into a shared space. Their semantic consistency is then quantified using the similarity function defined in Eq. (4).

4 $${sim}\left({S}_{i},{P}_{j}\right)={\left({h}_{s}\left({S}_{i}^{{cls}}\right)\right)}^{{{\rm{T}}}}{h}_{p}\left({P}_{j}^{{cls}}\right)$$

On this basis, we apply ${\mathrm{softmax}}$ normalization to construct probability distributions for inter-modal contrastive learning which are used for probability computation in the contrastive learning loss function:

5 $${S}^{s2p}\left({S}_{i},{P}_{j}\right) =\frac{\exp \left({sim}\left({S}_{i},{P}_{j}\right)/\tau \right)}{{\sum }_{q=1}^{Q}\exp \left({sim}\left({S}_{i},{P}_{q}\right)/\tau \right)}, \\ {S}^{p2s}\left({P}_{j}{,S}_{i}\right) =\frac{\exp \left({sim}\left({{P}_{j},S}_{i}\right)/\tau \right)}{{\sum }_{w=1}^{W}\exp \left({sim}\left({P}_{j},{S}_{w}\right)/\tau \right)}$$

Here, τ is the temperature coefficient that controls the smoothness of the softmax distribution, while W and Q denote the total numbers of SMILES and property samples used in the loss computation, respectively. Similarly, the probability distributions for intra-modal contrastive learning of s2s and p2p are defined as:

6 $$\begin{aligned}{S}^{s2s}\left({S}_{i},{S}_{j}\right)=\frac{\exp \left({sim}\left({S}_{i},{S}_{j}\right)/\tau \right)}{{\sum }_{w=1,w\ne i}^{W}\exp \left({sim}\left({S}_{i},{S}_{w}\right)/\tau \right)},\\ {S}^{p2p}\left({P}_{i},{P}_{j}\right)=\frac{\exp \left({sim}\left({P}_{i},{P}_{j}\right)/\tau \right)}{{\sum }_{q=1,q\ne i}^{Q}\exp \left({sim}\left({P}_{i},{P}_{q}\right)/\tau \right)\hfill}\end{aligned}$$

Unlike conventional contrastive learning that considers only exactly matched SMILES-property pairs as positive samples, we introduce a soft contrastive learning framework. The core idea is to replace binary partitioning with continuous similarity distributions, thereby avoiding mislabeling structurally similar variants with closely matching properties as negative samples, alleviating the “false negative” problem, and enabling more effective modeling of nonlinear structure-property relationships. Accordingly, we incorporate smooth soft labels generated by a momentum encoder across four tasks to suppress batch noise and enhance the stability and robustness of cross-modal alignment. Taking the s2p task as an example, we extract the global representations ${S}_{i}^{{cls},M}$ and ${P}_{j}^{{cls},M}$ of ${S}_{i}$ and ${P}_{j}$ using the momentum encoder, and construct a continuous soft-label distribution defined as:

7 $${\widetilde{y}}^{s2p}\left({S}_{i},{P}_{j}\right)=\frac{\exp \left({{sim}}^{M}\left({S}_{i},{P}_{j}\right)/\tau \right)}{{\sum }_{q=1}^{Q}\exp \left({{sim}}^{M}\left({S}_{i},{P}_{q}\right)/\tau \right)}$$

Here, ${{sim}}^{M}\left({S}_{i},{P}_{j}\right)={\left({h}_{S}\left({S}_{i}^{{cls},M}\right)\right)}^{\top}{h}_{P}\left({P}_{j}^{{cls},M}\right)$. We construct a hybrid target label by combining hard labels with soft labels generated by the momentum encoder:

8 $$\begin{array}{l}{y}^{s2p}\left({S}_{i},{P}_{j}\right)=\left(1-\alpha \right){\hat{y}}^{s2p}\left({S}_{i},{P}_{j}\right)+\alpha {\widetilde{y}}^{s2p}\left({S}_{i},{P}_{j}\right),\hfill\\ {\hat{y}}^{s2p}\left({S}_{i},{P}_{j}\right)=\left\{\begin{array}{cc}1, & {if}\,{S}_{i}\,{and}\,{P}_{j}\,{belong}\,{to}\,{the}\,{same}\,{molecule}\,\\ 0, & {otherwise}\hfill\end{array}\right.\end{array}$$

Here, α is a balancing coefficient that controls the relative strength of supervision from soft and hard labels. Accordingly, the s2p contrastive loss can be expressed as:

9 $${L}_{{CL}}^{s2p}={\sum }_{i=1}^{B}{\sum }_{j=1}^{Q}H\left({y}^{s2p}\left({S}_{i},{P}_{j}\right),\,{S}^{s2p}\left({S}_{i},{P}_{j}\right)\right)$$

Similarly, the contrastive losses for p2s, s2s, and p2p can be obtained, denoted as ${L}_{{CL}}^{p2s}$, ${L}_{{CL}}^{s2s}$ and ${L}_{{CL}}^{p2p}$ respectively. Therefore, the final contrastive learning loss is defined as:

10 $${L}_{{CL}}=\frac{1}{2}\left({L}_{{CL}}^{s2p}+{L}_{{CL}}^{p2s}\right)+\frac{1}{2}\left({L}_{{CL}}^{s2s}+{L}_{{CL}}^{p2p}\right)$$

*We design a conditional SMILES generation task* based on molecular properties and scaffold structures to enhance the controllability of molecular generation. The core objective of this task is to learn the mapping between conditional vectors and target molecular structures for directed generation under multiple constraints. For a molecule i, its property ${P}_{i}$ and scaffold ${{scaf}}_{i}$ are encoded into feature representations ${P}_{i}^{{en}}$ and ${s{caf}}_{i}^{{en}}$, which are concatenated into a conditional vector ${C}_{i}=[{P}_{i}^{{en}}{||}{s{caf}}_{i}^{{en}}]$. During decoding, ${C}_{i}$ is used as the Key and Value in the cross-modal decoder to provide global guidance for molecule generation, while the partially generated SMILES sequence is encoded by the structure encoder into a query representation that carries the current generation state. The cross-modal decoder leverages cross-attention to dynamically integrate conditional information with the generative state, thereby balancing property constraints with structural validity and progressively generating the desired SMILES sequence. The generation process follows an autoregressive mechanism, predicting the next token at each step by maximizing its conditional probability, with the loss function defined as:

11 $${L}_{S}={\sum }_{i=1}^{B}{\sum }_{n=0}^{N}\,H\left({y}_{n}^{S},{P}^{p2S}\left({s}_{n},|,{s}_{0:n-1},{C}_{i}\right)\right)$$

To enhance the model’s adaptability across different input conditions, we adopt a probabilistic mixture training strategy. At each training step, the model has a 50% probability of receiving only molecular property information as the generation condition ($C=[{P}^{{en}}]$), and a 50% probability of receiving both property and scaffold conditions jointly ($C=[{P}^{{en}}\parallel {{scaf}}^{{en}}]$). This mechanism not only improves the model’s robustness and adaptability under varying conditions but also enables it to flexibly adjust its generation strategy according to the completeness of the input conditions.

*The SMILES-based molecular property prediction task* aims to learn the mapping relationship from molecular structures to their properties. The structure encoder produces SMILES representation ${S}_{i}^{{en}}$ us as the Key and Value in the cross-modal decoder to guide property generation. Simultaneously, the prefix of the generated property sequence is encoded by the property encoder to produce the query representation. Similar to conditional molecular generation, this task adopts an autoregressive modeling strategy that not only captures the nonlinear relationships between structures and properties but also enables collaborative training with the molecular generation task through shared encoder parameters, thereby enhancing flexibility in multi-task prediction, with the loss function defined as:

12 $${L}_{P}={\sum }_{i=1}^{B}{\sum }_{m=0}^{M}\,H\left({y}_{m}^{p},{P}^{s2p}\left({p}_{m}|{p}_{0:m-1},{S}_{i}^{{en}}\right)\right)$$

Finally, we combine the five losses described above and perform end-to-end optimization of the model, and the total loss is defined as:

13 $$L={{L}_{{rec\_SMILES}}+{L}_{{rec\_property}}+L}_{{CL}}+{L}_{S}+{L}_{P}$$

### Downstream tasks

BiSP-GP has fully acquired the two core capabilities of conditional molecule generation and property prediction during the pretraining stage, enabling direct application in practical scenarios without additional fine-tuning. In molecular generation, most existing methods typically require separate training for different combinations of input properties, which limits their flexibility in real-world applications. In contrast, the pretrained BiSP-GP can flexibly combine the three properties used during pretraining with scaffold structures as input conditions to generate molecules satisfying all constraints, without needing retraining for each specific combination. For properties not intended to be controlled, the corresponding positions in the property sequence can simply be replaced with the special token [UNK], enabling unconstrained generation under missing conditions. When scaffold constraints are required, appending the scaffold SMILES expression to the end of the property sequence allows seamless integration of property and structure conditions. This input-level controllability enables BiSP-GP to handle diverse generation scenarios within a single model framework, substantially enhancing its practicality.

For other downstream tasks involving property classification or regression based on molecular SMILES representations, we adopt a fine-tuning architecture that retains the pretrained structure encoder as a molecular sequence representation extractor, followed by a multilayer perceptron (MLP) prediction head for task-specific classification or regression. Specifically, the structure encoder encodes the molecular sequence, and the global vector corresponding to the [CLS] token is extracted as the overall semantic representation of the molecule. This representation is then fed into the prediction head to obtain the final output.

### Dataset

We collected 20,000,000 SMILES representations of common molecules from the PubChem^60^ database for pretraining (Supplementary Table S10). Details of data preprocessing and standardization are provided in the Supplementary Note 2. To construct multi-dimensional supervision signals between molecular structures and properties, three key properties were computed for each compound using the RDKit^61^, including quantitative estimate of drug-likeness (QED)^62^, synthetic accessibility score (SAS)^63^, and logarithm of the partition coefficient (LogP)^64^. As shown in Supplementary Fig. S6, the distributions of these properties, along with molecular weight, indicate that the pretraining dataset covers a broad and chemically meaningful drug-like space. For downstream evaluation, we adopted the MoleculeNet benchmark datasets^50^ as well as two additional datasets, Malaria^51^ and CEP^52^ (Supplementary Table S10). A unified preprocessing pipeline was applied across all datasets to ensure consistency (Supplementary Note 2). The datasets were split using the scaffold splitter provided by DeepChem^65^, with an 8:1:1 ratio for training, validation, and test sets. Compared with random splitting, this scaffold-based splitting strategy imposes a more stringent evaluation setting and better reflects real-world extrapolation scenarios in drug discovery.

### Evaluation metrics

For molecular generation tasks, we employed four standard metrics provided by the Moses framework to evaluate the quality of generated molecules: validity, uniqueness, novelty, and internal diversity. For property-conditioned generation tasks, we additionally introduced MAD to assess the accuracy and stability of property control. In scaffold-constrained generation tasks, we used the ${Sim\_ratio}$, defined as the proportion of valid molecules with scaffold Tanimoto similarity above 0.8 to the input scaffold. For property prediction tasks, we adopted the coefficient of determination (${R}^{2}$) to evaluate the goodness of fit between predicted and ground-truth values. For the downstream classification and regression tasks, we used the area under the receiver operating characteristic curve (AUC) and the root mean square error (RMSE) as evaluation indicators, respectively. A detailed description of all evaluation metrics can be found in the Supplementary Note 3.

### Implementation details

Supplementary Table S11 summarizes the key hyperparameter configurations used during the pretraining and fine-tuning phases of the BiSP-GP model. In terms of model architecture, we adopted a dual-encoder design, where both the structure encoder and the property encoder employ a 6-layer BERT backbone *(hidden_size=*1024*, num_attention_heads=*6). The fusion decoder, structure decoder, and property decoder were all implemented using a 6-layer BERT variant equipped with cross-attention mechanisms. During pretraining, we used the AdamW optimizer with a learning rate of 5 × 10^−5^ and a weight decay coefficient of 0.02. Pretraining was conducted for 5 epochs with a batch size of 96. Notably, for contrastive learning tasks, we employed a large memory bank with a stack size of 12,288 to enhance negative sampling and set the temperature parameter τ=1. Additionally, the balancing coefficient α was set to 0.4 to regulate the relative supervision strength between soft and hard labels. During fine-tuning, the model was trained for 100 epochs with a batch size of 64.

### Reporting summary

Further information on research design is available in the Nature Portfolio Reporting Summary linked to this article.

## Supplementary information

Supplementary Information

Reporting summary

## Data Availability

The pretraining dataset was obtained from the PubChem public database (https://pubchem.ncbi.nlm.nih.gov/). The test scaffold and datasets used for SMILES-based property prediction tasks were sourced from the ZINC database (http://zinc.docking.org). The MoleculeNet datasets can be accessed via the DeepChem Python module (https://deepchem.io/) or downloaded directly from the official website (https://moleculenet.org/). The Malaria and CEP datasets are available from the HIPS neural-fingerprint repository (https://github.com/HIPS/neural-fingerprint).

## Code availability

The source code of BiSP-GP, along with experimental scripts and relevant datasets, has been made publicly available at https://github.com/xmubiocode/BiSP-GP and has been archived on Zenodo at https://zenodo.org/records/20115955.
