# Supplementary material for: Generative pretraining for drug molecule design with bidirectional structure-property optimization
Source: Commun Chem. 2026 Jun 8;9:298. doi: 10.1038/s42004-026-02093-5 (PMC13582974; doi:10.1038/s42004-026-02093-5)
Supplement: Supplementary file 1 [file 42004_2026_2093_MOESM1_ESM.pdf]

Generative pretraining for drug molecule design with bidirectional structure-property optimization

This document contains the following supplementary materials:

1. Supplementary Figures: S1-S6
2. Supplementary Tables: S1-S11
3. Supplementary Note 1: Molecular SMILES and property tokenization
4. Supplementary Note 2: Pretraining and downstream datasets preprocessing
5. Supplementary Note 3: Evaluation metrics

1. Supplementary Figures

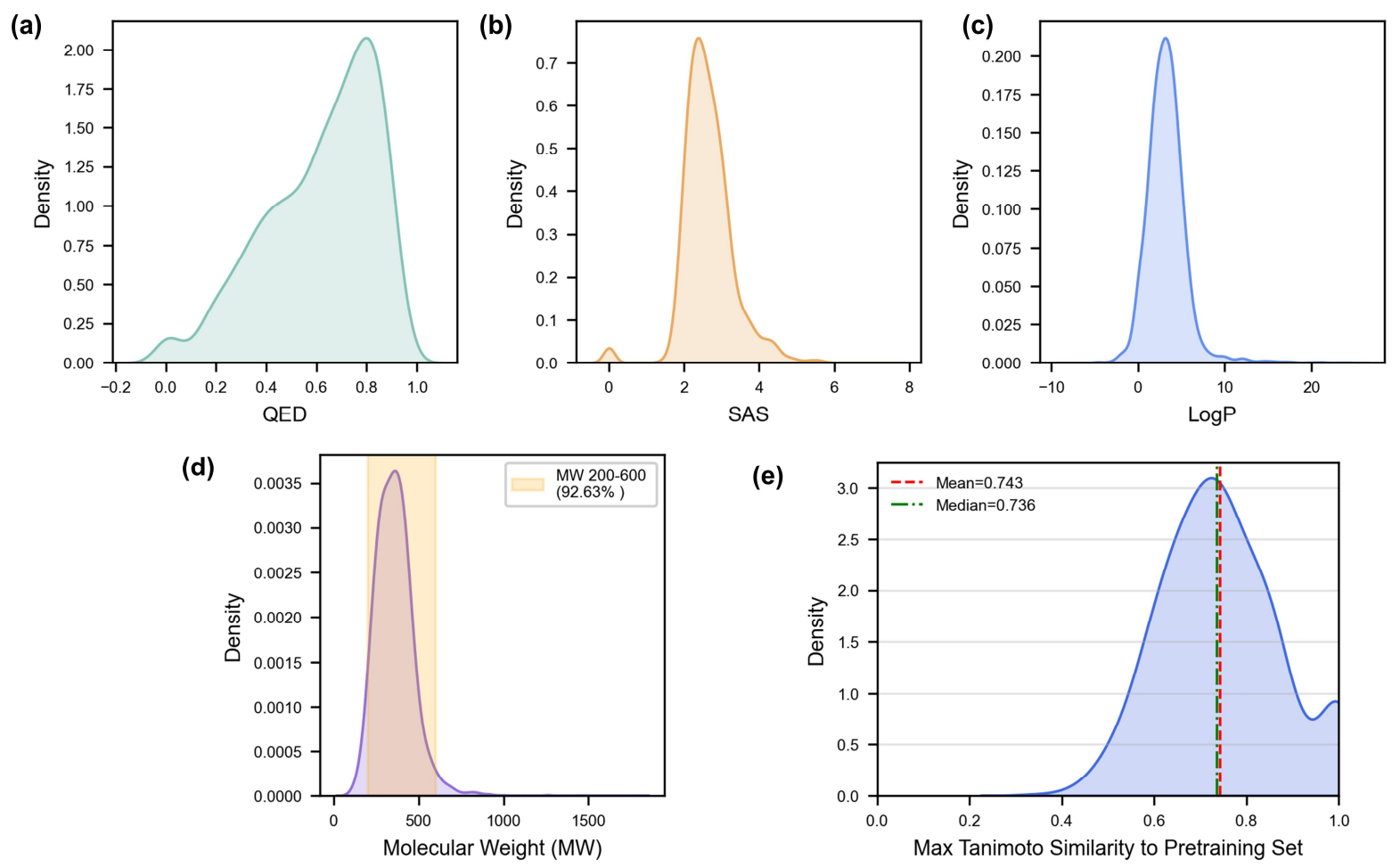

Figure S1. Property distributions and structural similarity analysis of molecules generated under the unconditional setting. (a-d) Distributions of four molecular properties (QED, SAS, LogP, and molecular weight) for the generated molecules. (e) Distribution of maximum Tanimoto similarity between generated molecules and the pre-training set, calculated based on Morgan fingerprints.

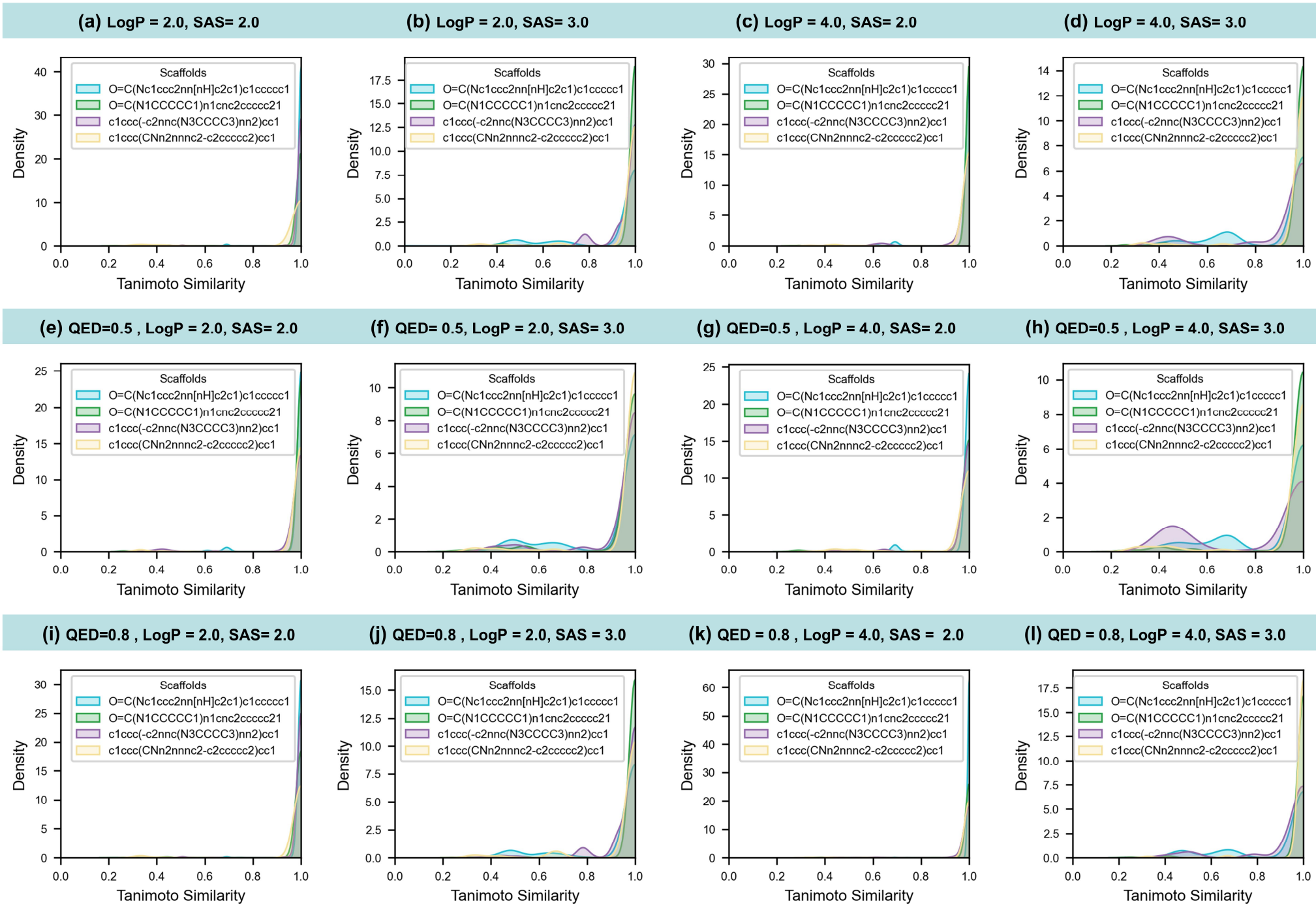

Figure S2. Tanimoto similarity distributions between the scaffolds of molecules generated by BiSP-GP and the corresponding input scaffolds under joint property and scaffold constraints. (a-d) Distributions of Tanimoto similarity under different LogP-SAS value combinations with scaffold constraints. (e-l) Distributions of Tanimoto similarity under different QED-LogP-SAS target combinations with scaffold constraints.

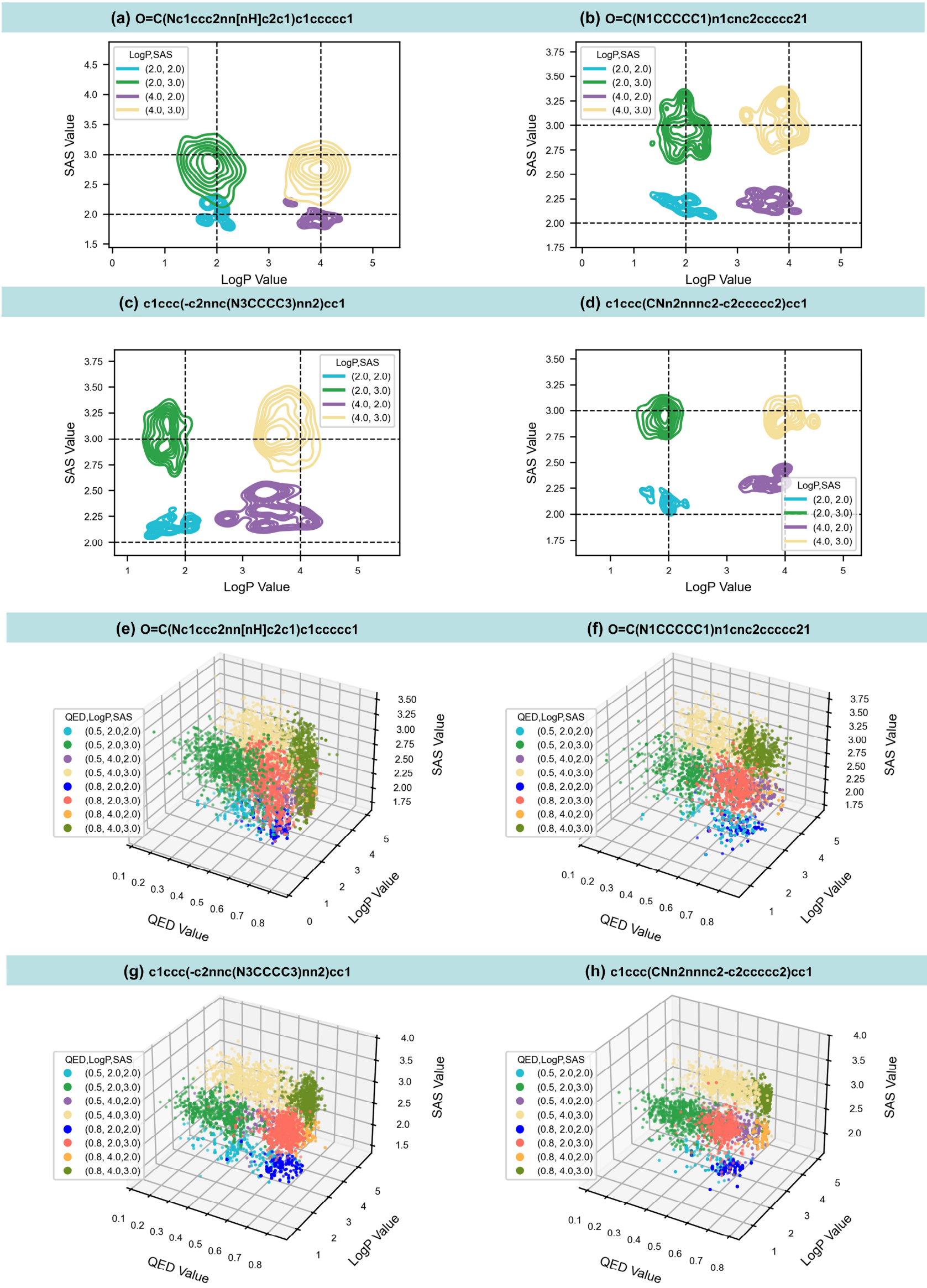

Figure S3. Property distributions of molecules generated by BiSP-GP under joint property and scaffold constraints. (a-d) Each of the four subfigures corresponds to one of the four input scaffolds, showing the property distributions of molecules generated under LogP-SAS target combinations. In the two-dimensional LogP-SAS distribution plots, different colored contour lines represent the density distribution of molecules generated under specified LogP and SAS target combinations. Tighter contour lines near the center indicate a high concentration of generated molecules in that region. Dashed intersections mark the corresponding target values, with the x-axis representing LogP and the y-axis representing SAS. (e-h) Each of the four subfigures corresponds to one of the four input scaffolds, showing the property distributions of molecules generated under QED-LogP-SAS target combinations. In the three-dimensional QED-LogP-SAS distribution plots, different colors represent distinct target property combinations, where the x-axis corresponds to QED, and the y-axis and depth-axis correspond to LogP and SAS, respectively.

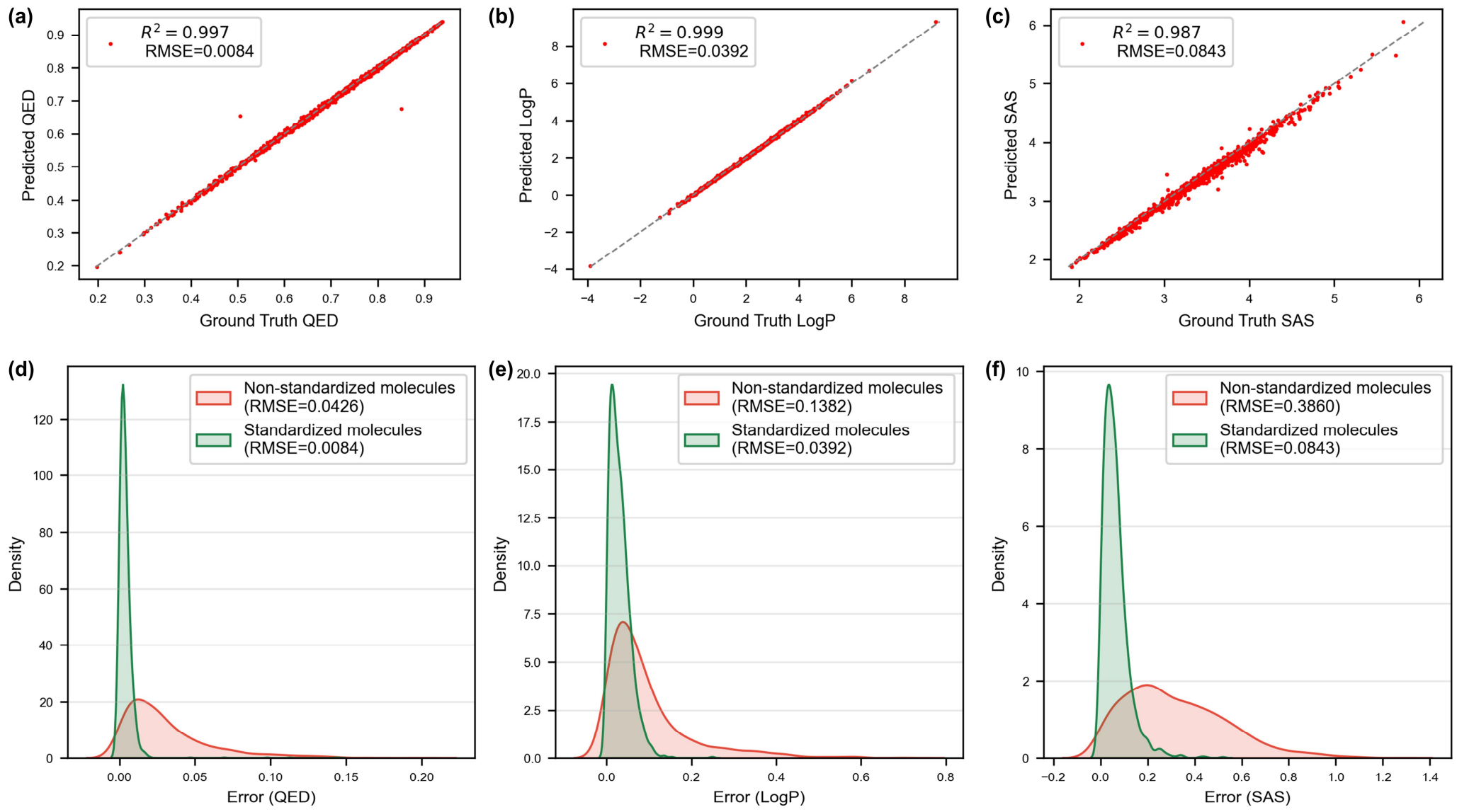

Figure S4. Comparison of property prediction performance and error distribution comparison between standardized and non-standardized molecules. (a-c) Prediction results of QED (a), LogP (b), and SAS (c) for 1000 molecules after standardization. The x-axis represents the ground truth values, the y-axis represents predicted values, and the dashed line denotes the y=x reference line. (d-f) Distribution of prediction errors for QED (d), LogP (e), and SAS (f), comparing standardized molecules with their non-standardized counterparts from the same set of 1,000 molecules.

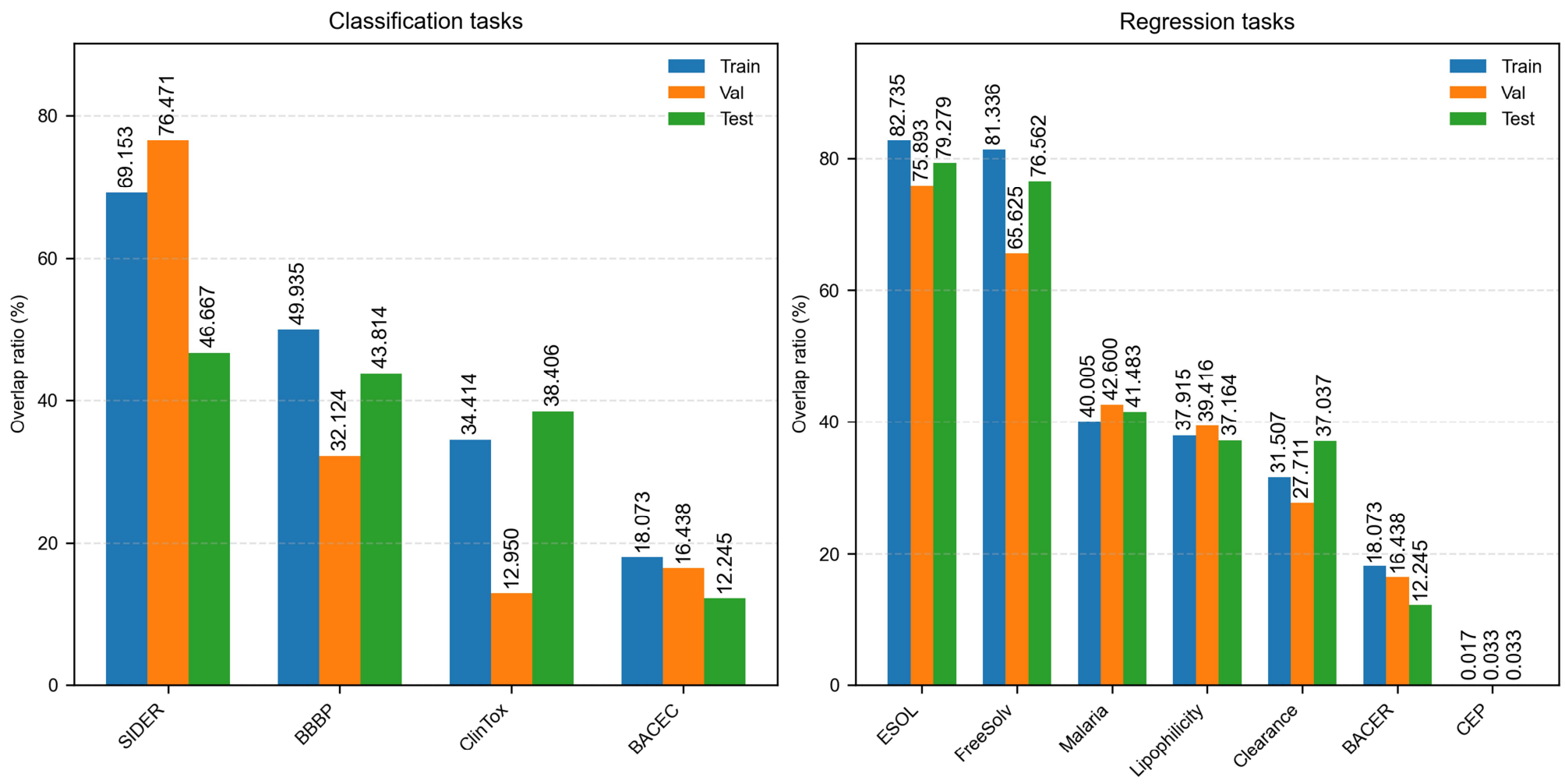

Figure S5. Overlap ratios between the pretraining dataset and downstream datasets across training, validation, and test splits, evaluated for both classification and regression tasks.

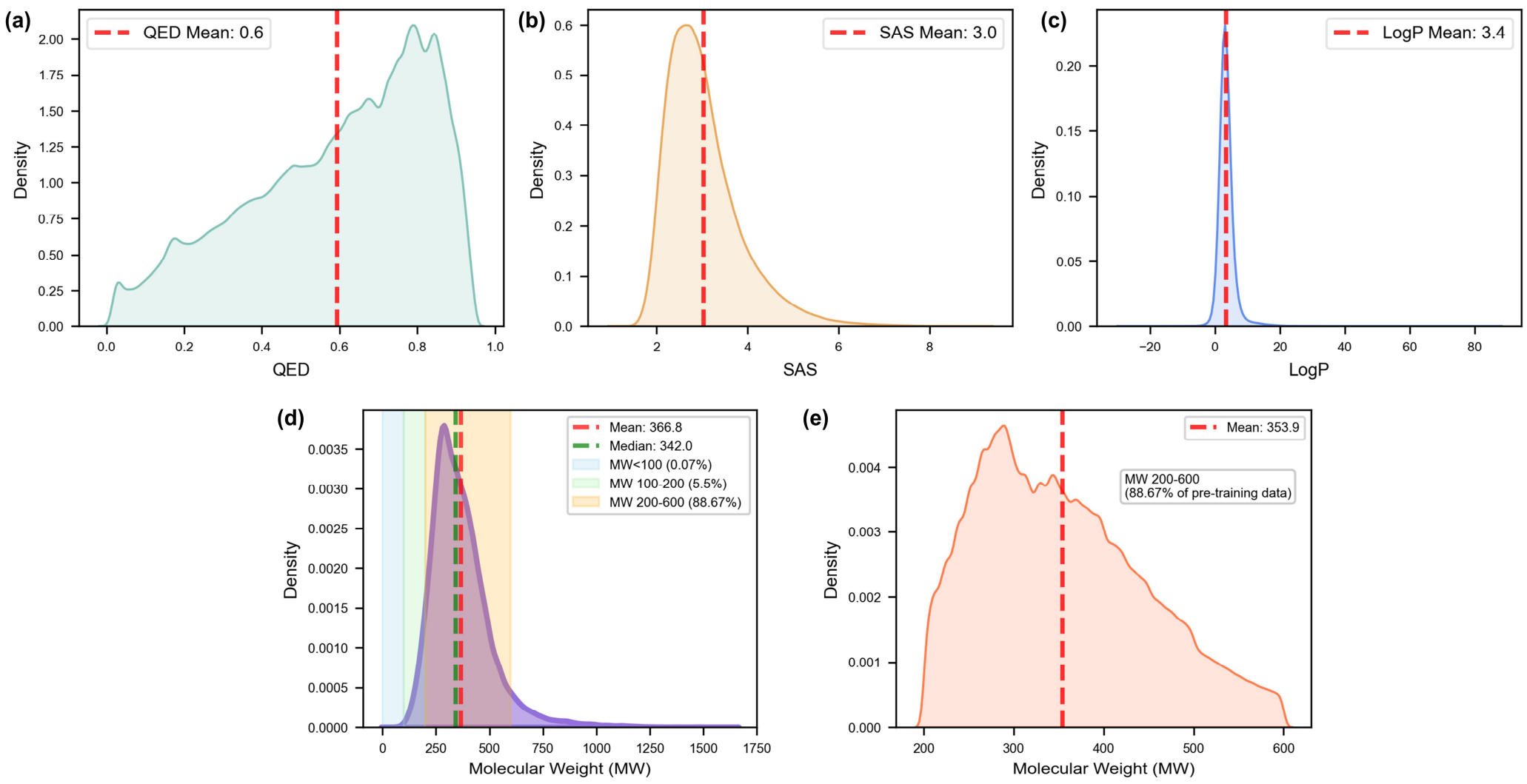

Figure S6. Property distributions in the pretraining dataset. Distributions of (a) QED, (b) SAS, (c) LogP, (d) molecular weight, and (e) molecular weight within the range of 200-600.

2. Supplementary Tables

Table S1. Molecular generation performance of BiSP-GP and baseline models under single-property constraints. All results are presented as mean ± standard deviation of the generation results across different property target values. Results marked with an asterisk (*) are directly taken from the original publications, while the others are reproduced using the released checkpoints of the baseline models.

Model	Val↑	Uni↑	Nov↑	IntD↑	MAD↓		
					QED	LogP	SAS
Scaffold-GGM*	0.978	0.864	0.993	-	-	0.29	-
CMGN	1.0±0.00	0.446±0.038	-	0.645±0.014	0.264±0.154	2.583±0.808	1.936±0.439
SPMM	0.949±0.059	0.997±0.003	0.985±0.012	0.834±0.013	0.172±0.062	5.467±0.130	-
BiSP-GP	0.989±0.006	0.999±0.001	0.915±0.010	0.871±0.014	0.066±0.023	0.280±0.029	0.149±0.047

Table S2. Molecular generation performance of BiSP-GP under multi-property constraints. All results are presented as mean ± standard deviation of the generation results across different property target value combinations.

Task	Val↑	Uni↑	Nov↑	MAD↓		
				QED	LogP	SAS
QED-LogP	0.983±0.009	0.996±0.003	0.924±0.014	0.066±0.022	0.284±0.048	-
QED-SAS	0.991±0.008	0.995±0.004	0.917±0.019	0.064±0.023	-	0.146±0.052
LogP-SAS	0.989±0.006	0.997±0.002	0.925±0.016	-	0.280±0.058	0.156±0.048
QED-LogP-SAS	0.987±0.011	0.988±0.010	0.927±0.025	0.064±0.026	0.281±0.061	0.158±0.054

Table S3. Regression performance of BiSP-GP and baseline models on downstream tasks. For each task, all models were trained (fine-tuned) employing four random seeds, with results reported as mean ± standard deviation (Std).

**Model**	**RMSE ± Std ↓**						
	**ESOL**	**LIPO**	**FreeSolv**	**BACE**	**Clearance**	**Malaria**	**CEP**
SMILES-Transformer	1.088±0.000	0.978±0.000	3.929±0.000	1.135±0.000	1.240±0.000	1.171±0.000	1.674±0.000
SPMM	0.910±0.063	0.716±0.009	1.982±0.018	1.069±0.035	49.103±0.455	1.311±0.010	3.447±0.089
SimSon	0.511±0.033	0.756±0.014	2.280±0.089	0.944±0.130	0.921±0.024	1.097±0.007	1.465±0.030
BiSP-GP (Ours)	0.450±0.011	0.608±0.025	0.513±0.077	0.753±0.040	0.955±0.016	1.060±0.014	1.369±0.022

Table S4. Regression performance of BiSP-GP and baseline models on downstream tasks. Results are reported as RMSE with 95% confidence intervals. p-values were computed using bootstrap's test based on averaged predictions across random seeds.

**Model**	**RMSE (95% CI) ↓**						
	**ESOL**	**LIPO**	**FreeSolv**	**BACE**	**Clearance**	**Malaria**	**CEP**
SMILES-Transformer	1.088 (0.928, 1.228)	0.978 (0.884, 1.069)	3.929 (3.041, 4.896)	1.135 (0.985, 1.296)	1.240 (1.049, 1.427)	1.171 (1.111, 1.240)	1.674 (1.582, 1.770)
p	<0.01	<0.01	<0.01	<0.01	<0.01	<0.01	<0.01
SPMM	0.888 (0.774, 1.014)	0.693 (0.642, 0.747)	1.945 (1.441, 2.431)	1.041 (0.890, 1.192)	48.640 (39.830, 56.777)	1.258 (1.181, 1.346)	3.065 (2.757, 3.375)
p	<0.01	<0.01	<0.01	<0.01	<0.01	<0.01	<0.01
SimSon	0.454 (0.390, 0.517)	0.718 (0.668, 0.769)	2.229 (1.741, 2.719)	0.886 (0.784, 0.988)	0.893 (0.768, 1.003)	1.085 (1.021, 1.155)	1.412 (1.327, 1.492)
p	<0.05	<0.01	<0.01	<0.01	0.20	<0.01	<0.01
BiSP-GP (Ours)	0.396 (0.344, 0.445)	0.577 (0.526, 0.630)	0.442 (0.358, 0.524)	0.706 (0.629, 0.792)	0.936 (0.784, 1.083)	1.023 (0.958, 1.091)	1.244 (1.131, 1.348)

Table S5 Classification performance of BiSP-GP and baseline models on downstream tasks. All models were trained (fine-tuned) employing four random seeds, with results reported as mean ± standard deviation (Std).

Model	AUC ± Std ↑			
	BACE	BBBP	Clintox	SIDER
Random chance predictions	0.508±0.032	0.527±0.025	0.432±0.094	0.503±0.017
y-scrambling	0.496±0.046	0.535±0.033	0.500±0.054	0.490±0.018
1-nearest neighbour	0.791±0.000	0.642±0.000	0.627±0.000	0.558±0.000
SMILES-Transformer	0.806±0.000	0.684±0.000	0.687±0.000	0.580±0.000
SPMM	0.798±0.012	0.757±0.009	0.827±0.021	0.627±0.007
SimSon	0.751±0.064	0.768±0.023	0.733±0.065	0.567±0.010
BiSP-GP (Ours)	0.817±0.004	0.772±0.002	0.890±0.006	0.622±0.006

Table S6. Classification performance of BiSP-GP and baseline models on downstream tasks. Results are reported as AUC with 95% confidence intervals. p-values were computed using DeLong's test based on averaged predictions across random seeds.

Model	BACE		BBBP		Clintox		SIDER	
	AUC (95% CI)	*p*	AUC (95% CI)	*p*	AUC (95% CI)	*p*	AUC (95% CI)	*p*
Random chance predictions	0.537 (0.442, 0.632)	<0.01	0.560 (0.479, 0.641)	<0.01	0.382 (0.251, 0.507)	<0.01	0.506 (0.479, 0.535)	<0.01
y-scrambling	0.505 (0.408, 0.601)	<0.01	0.548 (0.466, 0.629)	<0.01	0.510 (0.416, 0.602)	<0.01	0.483 (0.456, 0.508)	<0.01
1-nearest neighbour	0.791 (0.726, 0.855)	0.260	0.642 (0.583, 0.700)	<0.01	0.627 (0.472, 0.797)	<0.01	0.558 (0.526, 0.593)	<0.01
SMILES-Transformer	0.806 (0.735, 0.876)	0.471	0.684 (0.609, 0.758)	<0.01	0.687 (0.511, 0.846)	<0.01	0.580 (0.537, 0.623)	<0.05
SPMM	0.803 (0.731, 0.875)	0.134	0.757 (0.690, 0.824)	0.433	0.835 (0.672, 0.965)	0.091	0.632 (0.574, 0.684)	0.06
SimSon	0.787 (0.713, 0.860)	0.160	0.785 (0.719, 0.851)	0.657	0.804 (0.706, 0.888)	<0.05	0.589 (0.552, 0.626)	0.377
BiSP-GP (Ours)	0.830 (0.764, 0.897)	-	0.775 (0.709, 0.841)	-	0.892 (0.784, 0.987)	-	0.634 (0.586, 0.684)	-

Table S7. Experimental results of BiSP-GP and its two variants in three molecular generation tasks.

Constraints	Model	Val ↑	Uni ↑	Nov ↑	MAD ↓		
					QED	LogP	SAS
Unconditioned	BiSP-GP value emb	0.948±0.003	0.999±0.001	0.904±0.003	-	-	-
	BiSP-GP w/o CL	0.946±0.005	0.999±0.001	0.925±0.009	-	-	-
	BiSP-GP	0.986±0.004	0.999±0.001	0.926±0.004	-	-	-
Single Property	BiSP-GP value emb	0.969±0.017	0.997±0.002	0.909±0.023	0.112±0.04	0.833±0.020	0.325±0.072
	BiSP-GP w/o CL	0.974±0.014	0.994±0.001	0.914±0.010	0.086±0.027	0.387±0.019	0.194±0.053
	BiSP-GP	0.989±0.006	0.999±0.002	0.915±0.010	0.066±0.023	0.280±0.029	0.149±0.047
Multiproperty	BiSP-GP value emb	0.973±0.016	0.997±0.004	0.907±0.025	0.126±0.037	0.787±0.126	0.350±0.130
	BiSP-GP w/o CL	0.976±0.018	0.995±0.007	0.919±0.023	0.085±0.028	0.409±0.041	0.188±0.051
	BiSP-GP	0.988±0.009	0.994±0.005	0.923±0.056	0.065±0.026	0.281±0.056	0.153±0.051

Table S8. Experimental results of BiSP-GP and its two variants on SMILES-based property prediction tasks.

Model	R^2 ↑		
	QED	LogP	SAS
BiSP-GP value emb	0.878	0.801	0.908
BiSP-GP w/o CL	0.988	0.990	0.954
BiSP-GP	0.999	0.997	0.987

Table S9. Experimental results of BiSP-GP and its two variants on 11 downstream fine-tuning tasks, including seven regression and four classification tasks.

Task & Metric	Dataset	BiSP-GP value emb	BiSP-GP w/o CL	BiSP-GP
Regression (RMSE ↓)	ESOL	0.513±0.025	0.449±0.023	0.450±0.011
	LIPO	0.654±0.015	0.613±0.013	0.608±0.025
	FreeSolv	0.531±0.055	0.575±0.031	0.513±0.077
	BACE	0.740±0.044	0.798±0.102	0.753±0.040
	Clearance	0.999±0.046	0.974±0.034	0.955±0.016
	Malaria	1.058±0.009	1.069±0.012	1.060±0.014
	CEP	1.429±0.027	1.406±0.016	1.369±0.022
Classification (AUC ↑)	BBBP	0.775±0.006	0.777±0.007	0.817±0.004
	BACE	0.767±0.014	0.711±0.014	0.772±0.002
	Clintox	0.869±0.003	0.875±0.007	0.890±0.006
	SIDER	0.567±0.008	0.607±0.012	0.622±0.006

Table S10. Dataset and evaluation settings for pretraining, generation, property prediction and downstream tasks.

Category	Task Type	Dataset	Training Set	Validation Set	Test Set
**Pretraining**	Structure-property generative pretraining	PubChem	20M	-	-
**Molecule Generation**	Unconditional generation	-	-	-	1000 generated molecules
	Property-constrained generation	-	-	-	1000 generated molecules per condition
	Scaffold-constrained generation	ZINC (scaffold set)	-	-	1000 generated molecules per scaffold (100 scaffolds)
**Property Prediction**	SMILES-to-property generation (QED, LogP, SAS)	ZINC	-	-	1000 molecules
**Downstream Task**	Classification	BBBP	1550	194	193
	Classification	BACE	1173	147	146
	Classification	Clintox	1109	139	139
	Classification	SIDER	1085	136	136
	Regression	ESOL	892	111	112
	Regression	LIPO	3280	410	410
	Regression	FreeSolv	509	64	64
	Regression	BACE	1172	147	147
	Regression	Clearance	656	82	83
	Regression	Malaria	7993	999	1000
	Regression	CEP	23972	2997	2997

Table S11. Main hyperparameters and settings of the BiSP-GP model.

Hyperparameters	Setting
Pretrain Epoch	5
Pretrain Batch Size	96
Learning rate	5e-5
Number of encoder and decoder layers	6
hidden_size	1024
Stack size	12288
τ	1
α	0.4
Finetuning Epoch	100
Finetuning Batch Size	64

3. Supplementary Note 1: Molecular SMILES and property tokenization

Tokenization of Molecular SMILES. In this study, we adopt SMILES (Simplified Molecular Input Line Entry System) strings as the standardized representation of molecular structures. To enhance semantic understanding of SMILES sequences, we employ the Byte Pair Encoding (BPE)[1,2] algorithm for molecular tokenization. As a statistical subword segmentation method, BPE iteratively merges frequent character pairs to construct a domain-specific vocabulary containing 367 chemical subwords. In the molecular context, this algorithm automatically identifies and encodes chemically meaningful substructures, such as hydroxyl groups (-OH), carbonyl groups (C=O), and aromatic rings (c1ccccc1). Compared with traditional character-level tokenization, BPE not only significantly reduces sequence length and improves computational efficiency, but also preserves the integrity of chemical substructures, enhances the recognition of local molecular features, and better captures recurring patterns and structural regularities within molecules. For sequence representation, we use two special tokens, [CLS] and [SEP], to mark the start and end of each SMILES sequence, respectively. Specifically, the [CLS] token aggregates global information from the entire molecular sequence via a bidirectional attention mechanism. Its embedding serves as a compact representation of molecular structure, providing a unified feature interface for downstream tasks such as contrastive learning, classification, and regression. This design not only preserves the flexibility of sequence modeling but also effectively captures holistic structural features of molecules, thereby establishing a solid foundation for multi-task learning.

Tokenization of Molecular Property. In contrast to traditional methods that model target variables as deterministic functions of inputs, which risk introducing excessively rigid assumptions, we introduce a generalized sequence-modeling framework that encodes continuous molecular property values into structured character sequences. As defined by the Grammar 1, we first employ a regular expression-based parser to decompose raw numeric values into four semantic components, sign (*Sign*),

integer part (*IntegerPart*), decimal point (*DecimalPoint*), and decimal part (*DecimalPart*). These components are then transformed into a sequence of discrete tokens. Specifically, each digit is represented as "$\_Value\_Position\_$", where $Value$ is the digit (0-9), and $Position$ is its positional index. For the integer digits (*DigitToken_k*), positional indices start from 0 at the units place and increase upward; for the fractional digits (*DigitToken_j*), positional indices start from -1 at the first digit after the decimal point and decrease downward. The *Sign* is encoded as "_+_" or "_-_", and the *DecimalPoint* as "_._" For example, the property value -0.23 is represented as the token sequence "$\_-\_\_0\_0\_\_.\_\_2\_-1\_\_3\_-2\_$". To construct the complete serialized property representation, we draw inspiration from SMILES encoding. A special token [CLS] is inserted at the beginning of each property sequence, and the sequence is terminated with [SEP]. This representation ensures clearer and more standardized decoding of property values while preserving full decimal precision and positional information. It also enables the model to effectively recognize and distinguish the positional weights of different digits, thereby enhancing its ability to interpret and represent molecular property features.

Grammar 1. Property sequence serialization

<PropertySequence> → [CLS] <Property> {<Property>} [SEP]
<Property> → <PropertyIdentifier> <PropertyValue>
<PropertyIdentifier> → <QED> | <LogP> | ...
<PropertyValue > → <Sign> <IntegerPart> <DecimalPoint> <DecimalPart>
<Sign> → _+_ | _-_
<IntegerPart> → <DigitToken_k> {<DigitToken_k>}
<DecimalPoint> → _._
<DecimalPart> → <DigitToken_j> {<DigitToken_j>}
<DigitToken_k> → _<Value>_<Position_k>_
<DigitToken_j> → _< Value >_<Position_j>_
<Value> → 0 | 1 | 2 | 3 | 4 | 5 | 6 | 7 | 8 | 9
<Position_k> → 0 | 1 | 2 |⋯
<Position_j> → −1 | −2 | −3 |⋯

4. Supplementary Note 2: Pretraining and downstream datasets preprocessing

1) Pretraining dataset preprocessing

To ensure data consistency and minimize representational variability, all molecular structures were initially converted to canonical SMILES using RDKit. Stereochemical annotations were subsequently removed to direct the model's focus toward scaffold-level structural features and to bolster the robustness of large-scale pretraining. Standard sanitization procedures, including valence checks and aromaticity perception, were applied to guarantee chemical validity, and any invalid or unparsable SMILES were discarded.

2) Downstream dataset preprocessing

To ensure consistency and reliability in downstream evaluation, all molecular datasets were processed using a unified RDKit-based preprocessing pipeline. First, molecules were standardized by removing salts and retaining the largest organic fragment. Tautomer canonicalization was subsequently applied to unify different tautomeric forms into a single representative structure. As stereochemical annotations in public datasets are often incomplete or unreliable, stereochemical information was removed to generate consistent achiral SMILES representations, thereby precluding ambiguity during model training. Second, deduplication was performed based on the first 14 characters of the InChIKey (connectivity layer) computed from the desalted molecules. This approach guarantees that compounds sharing identical 2D connectivity are uniquely represented, irrespective of stereochemical variations or salt forms, effectively mitigating redundancy arising from poorly annotated stereochemistry.

5. Supplementary Note 3: Evaluation metrics

For molecular generation tasks. We adopted four fundamental metrics provided by the Moses framework[3], a standardized benchmarking platform for evaluating molecular generative models. These metrics include Validity, Uniqueness, Novelty, and Internal Diversity, with their calculation formulas defined as follows.

Validity (Val) measures the proportion of generated molecules that successfully pass chemical structure validation, reflecting the baseline chemical feasibility of the generative model.

$$Validity = \frac{|S_{valid}|}{|S_{gene}|} \quad (1)$$

where S_{valid} denotes the set of valid molecules, S_{gene} represents the set of all generated molecules, and $|\cdot|$ represents the cardinality of a set, i.e., the number of molecules it contains.

Uniqueness (Uni) measures the proportion of non-redundant molecules among all valid generated molecules, reflecting the model's ability to explore the chemical structural space. It is defined as follows:

$$Uniqueness = \frac{|Set(S_{valid})|}{|S_{valid}|} \quad (2)$$

where $Set(S_{valid})$ denotes the deduplicated set obtained from the collection of valid molecules.

Novelty (Nov) measures the proportion of valid generated molecules that contain unique structures not present in the training set, thereby evaluating the model's ability to generate novel compounds.

$$Novelty = \frac{|Set(S_{valid}) \setminus S_{train}|}{|Set(S_{valid})|} \quad (3)$$

where S_{train} denotes the set of molecules in the training dataset, and $Set(S_{valid}) \setminus S_{train}$ represents the subset of valid unique molecules that do not appear in the training set.

Internal Diversity (IntD) measures the structural variability among valid generated molecules. It reflects the extent of chemical space coverage and the novelty of generated structures, and is typically calculated based on pairwise molecular

similarities.

$$IntD = 1 - \frac{1}{|Set(S_{valid})|^2} \sum_{s_i, s_j \in Set(S_{valid})} Tc(s_i, s_j) \tag{4}$$

where $Tc(s_i, s_j)$ denotes the Tanimoto coefficient computed between the Morgan fingerprints (radius=2, 2048 bits) of molecules s_i and s_j, quantifying the similarity between their molecular representations. It is defined as follows:

$$TC(s_i, s_j) = \frac{\left|f_{s_i} \cap f_{s_j}\right|}{\left|f_{s_i}\right| + \left|f_{s_j}\right| - \left|f_{s_i} \cap f_{s_j}\right|} \tag{5}$$

where f_{s_i} and f_{s_j} denote the Morgan fingerprint sets of molecules s_i and s_j, respectively. A higher IntD value indicates greater structural variability among the generated molecules, reflecting better diversity in the model's outputs. Conversely, an IntD value approaching 0 suggests that the generated molecules are overly concentrated within a limited set of structures.

For property-based molecular generation tasks. We employ the mean absolute deviation (MAD) to assess the accuracy and robustness of property control in generated molecules.

$$MAD = \frac{1}{n} \sum_{i=1}^{n} |y - \hat{y}_i| \tag{6}$$

where y denotes the input property value, $\hat{y}_i$ represents the property value of the i-th generated molecule, and n is the total number of valid generated molecules.

For scaffold-constrained molecular generation tasks. We introduce the scaffold similarity ratio (Sim_ratio) as a key evaluation metric. It is defined as the proportion of valid generated molecules whose scaffolds exhibit a Tanimoto similarity greater than 0.8 with the input scaffold. Here, the Tanimoto similarity (also referred to as the Tanimoto coefficient) is used to quantify the similarity between molecular fingerprints. The *Sim_ratio* quantifies the model's ability to preserve designated structural fragments, and its calculation is given as follows:

$$Sim\_ratio = \frac{\left|\{s \epsilon S_{valid} | TC(sf_{input}, mol\_sf(s)) \geq 0.8\}\right|}{|S_{valid}|} \tag{7}$$

where $mol\_sf(s)$ refers to the scaffold structure extracted from a valid generated

molecule s, and sf_{input} denotes the input scaffold.

For the SMILES-to-property prediction task. We adopt the coefficient of determination (R^2) as the primary evaluation metric, which measures the goodness of fit between predicted and true values.

$$R^2 = 1 - \frac{\sum_{i=1}^{n}(y_i - \hat{y}_i)^2}{\sum_{i=1}^{n}(y_i - \overline{y})^2} \tag{8}$$

where y_i denotes the true property value of the i-th test molecule, $\hat{y}_i$ represents the predicted property value of the i-th molecule, and $\overline{y}$ is the mean of the true property values across all molecules.

For downstream classification tasks. We use the area under the receiver operating characteristic curve (AUC) as the evaluation metric, which assesses the model's discriminative ability.

$$AUC = \int_0^1 TPR(FPR)dFPR \tag{9}$$

where TPR (true positive rate) denotes the proportion of positive samples correctly predicted as positive, and FPR (false positive rate) refers to the proportion of negative samples incorrectly predicted as positive.

For downstream regression tasks. We employ the root mean square error (RMSE) as the evaluation metric, which quantifies the deviation between predicted and true values.

$$RMSE = \sqrt{\frac{1}{n}\sum_{i=1}^{n} (y_i - \hat{y}_i)^2} \tag{10}$$

Supplementary References

1 Zouhar, V. *et al.* A Formal Perspective on Byte-Pair Encoding. *Findings of the Association for Computational Linguistics: ACL 2023.* 598-614 (2023).

2 Götz, L., Kollovieh, M., Günnemann, S. & Schwinn, L. Byte Pair Encoding for Efficient Time Series Forecasting. *arXiv preprint arXiv:2505.14411.* (2025).

3 Polykovskiy, D. *et al.* Molecular sets (MOSES): a benchmarking platform for molecular generation models. *Frontiers in pharmacology.* **11**, 565644 (2020).
